# Oleanolic acid combined with aspirin plays antitumor roles in colorectal cancer via the Akt/NFκB/IκBα/COX2 pathway

**DOI:** 10.1038/s41420-024-02223-9

**Published:** 2024-12-18

**Authors:** Yulv Zhou, Shengnan Lin, Xinzhu Zhong, Fang Huang, Jinxiang Huang, Luning Xu

**Affiliations:** 1 Department of Chinese Medicine and Anorectology, Sanming First Hospital, Affiliated Hospital of Fujian Medical University, Sanming City, Fujian Province China; 2 Department of Clinical Pharmacy, Sanming First Hospital, Affiliated Hospital of Fujian Medical University, Sanming City, Fujian Province China; 3 Department of Neurosurgery, The First Affiliated Hospital of Naval Medical University (Changhai Hospital), Naval Medical University, Shanghai, China

**Keywords:** Cancer, Cell signalling

## Abstract

Among the common malignancies, colorectal cancer (CRC) is often resistant to chemotherapy because of drug resistance and severe toxicity. Currently, aspirin is one of the most promising CRC chemopreventive drugs, both for primary prevention and for reducing the chance of recurrence and metastasis following radical surgery in patients with early-stage CRC. Oleanolic acid is a potential antineoplastic drug that has an antagonistic effect on many kinds of tumors. Network pharmacology, molecular docking, and in vitro experiments were performed to investigate whether OA combined with aspirin can enhance the anticancer effects of aspirin. As indicated by the network pharmacology results, oleanolic acid and aspirin can regulate multiple signaling pathways through multiple target proteins, including NFκB1\IκBα\PTGS2\MAPK3\PIK3CA. A series of cellular experiments demonstrated for the first time that oleanolic acid synergistically enhances aspirin to inhibit the proliferation and invasion of HCT116 and HT29 cells and induce S-phase arrest by regulating Akt/NFκB/IκBα/COX2 signaling pathway, thus synergistically enhancing the ability of aspirin to promote apoptosis of colorectal cancer cells. This study provides a novel approach to the use of fresh medications for the treatment of colorectal cancer and offers a theoretical foundation for the potential creation of aspirin derivatives based on oleanolic acid.

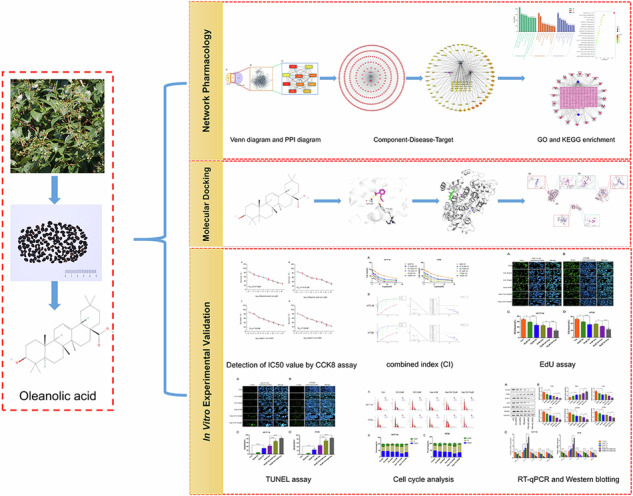

## Introduction

According to the most recent study, colorectal cancer (CRC) is one of the most common cancers in the world, with the third-highest incidence and second-highest fatality rate among all cancers [1]. For CRC, the standard treatment options are surgery, chemotherapy, and radiotherapy. The majority of patients are clinically diagnosed with advanced CRC and are only eligible for chemotherapy because of the disease’s sneaky nature and vague symptoms. Furthermore, even CRC patients who undergo major resection should be followed up with chemotherapy and radiation treatment. In contrast, conventional radiotherapy regimens for CRC not only have low cure rates but are also associated with severe side effects, drug resistance, and severe toxicity, limiting their effectiveness [2, 3]. There are currently no effective anti-CRC drugs with minimal side effects currently available. Therefore, there is an urgent need to find new, effective anti-CRC drugs with minimal side effects. Recent studies revealed aspirin to be a promising chemopreventive drug for CRC, not only as a primary preventive measure but also as a means of reducing recurrence and metastasis risk after radical surgery [4]. The long-term administration of the drug can cause side effects, such as gastrointestinal bleeding, that limit its use in clinical settings [5]. researchers have Encouragingly, a large number of natural compounds as potential anticancer agents that exhibit cytotoxic and antiproliferative properties [6]. Phytochemical compounds have received great attention in chemoprevention due to their low toxicity and low cost [7], and a variety of phytochemicals have been shown to have chemopreventive effects against CRC in numerous studies. Therefore, phytochemicals are expected to be a new option for CRC prevention and treatment [8].

**Fig. 1:**
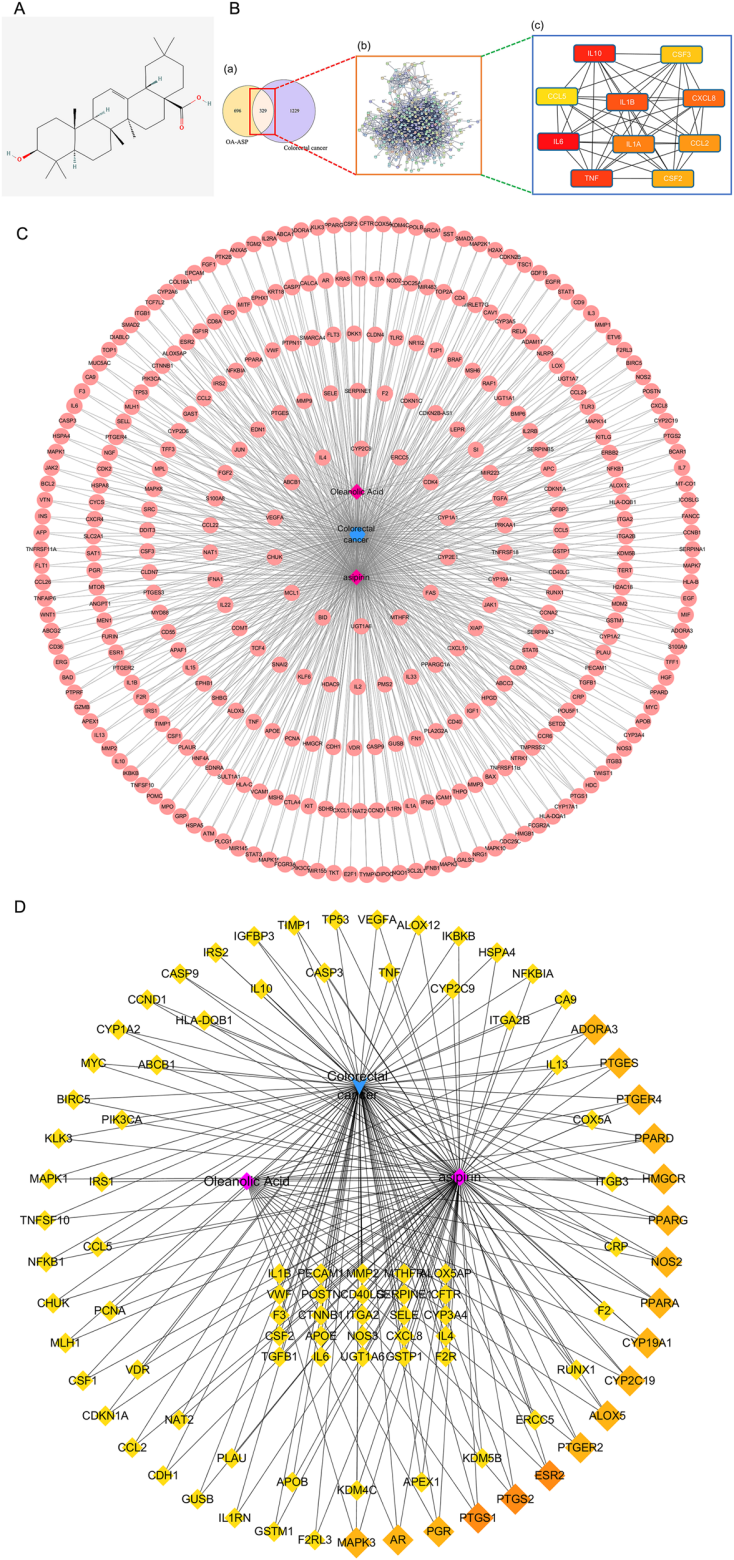
Network pharmacologic results of oleanolic acid and aspirin in the treatment of colorectal cancer. **A** the structure of oleanolic acid; **B** A Venn diagram showing the relationship between oleanolic acid, aspirin, and colorectal cancer targets. ((a): Venn diagram of target intersection of OA-Asp and colorectal cancer; (b and c): PPI diagram of OA-Asp and colorectal cancer.); **C** Oleanolic acid-aspirin-colorectal cancer-target network. **D** Oleanolic acid, aspirin-colorectal cancer-target network in the top 100 degrees of freedom.

A pentacyclic triterpenoid, oleanolic acid (OA: 3-hydroxy-olea-12-en-28-oic acid; Fig. 1A), can be found in a wide range of medicinal and edible plants. It has pharmacological effects such as antioxidant [9], anti-inflammatory [10], antiulcer [11], antibacterial [12], and antiviral [13], various tumor types are also affected by its antagonistic effects [14, 15]. A variety of tumor cells can be induced to undergo apoptosis when exposed to OA, such as those found in gliomas [14], lung cancers, pancreatic cancers, cervical cancer [16], ovarian cancer cells [17], and many other tumor cells with antiproliferative effects. The effects of OA on colon cancer cell proliferation were previously shown to be dose-dependent [18, 19]. Another study showed that OA induced oxidative stress in HCT116 cells and activated apoptosis [20]. Hu et al. also demonstrated that OA stimulated AMPK-mTOR signaling to induce autophagy and apoptosis in colon cancer cells [21]. A number of Phase I clinical trials have demonstrated the potent antitumor activity of OA and its derivatives in advanced solid tumors with limited toxicity [22, 23]. In addition to enhancing chemotherapeutic activity, OA protects normal cells from harmful effects of chemotherapeutic agents and enhances the chemotherapeutic activity of camptothecin-11 [24]. The use of OA for the treatment of CRC could thus be very promising.

A network pharmacology approach examines diseases, human biological systems, and drug targets from a network perspective by combining bioinformatics, high-throughput histological analysis, and computer technology [25, 26]. With its unique advantage of providing new ideas and directions for studying complex traditional Chinese medicine systems (TCM), network pharmacology has become widely used in TCM systems. An important part of the process of finding new drugs is predicting the affinity and binding pattern of small molecules to large molecules using a computer program known as molecular docking [27]. From the point of view of network pharmacology and molecular docking, systematic classification of database resources from the point of view of database resources is a potential research direction. However, it is necessary to verify the findings in cellular or animal experiments due to the virtual nature of network pharmacology and molecular docking.

We hypothesized that combined treatment with OA and aspirin might enhance the anti-CRC activity of aspirin since both are anti-CRC agents. Through network pharmacology and molecular docking, we explored the underlying mechanisms of OA and aspirin against CRC. Next, we examined HT-29 and HCT-116 CRC cells for their biological behavior and investigated the mechanism of their anti-CRC action when combined with aspirin. To provide a theoretical basis for improving the antitumor effect of aspirin, we conducted a series of in vitro experiments, including CCK8 colorimetric assays, EdU assays, TUNEL assays, flow cytometry, and western blots.

## Results

### Acquisition of active ingredients, disease targets and PPI topology analysis

By searching databases, we found 78 targets associated with OA, 978 targets associated with aspirin, 1494 targets associated with CRC found in the Gene Cards database and 158 from the OMIM database. It was possible to obtain 1558 CRC-related targets by combining the two and removing the repetitive values. It can be seen in Fig. 1B(a) that disease targets intersected with component targets, resulting in 329 common targets. Among the intersecting targets, OA-enhanced aspirin was considered a potential target for the treatment of CRC. Figure 1C shows a network of drug-disease targets constructed using Cytoscape 3.2.1. With 327 nodes and 656 edges, the network consists of 327 nodes. The cytoHubba plug-in is used to select the target genes with the first 100 degrees of freedom of nodes to construct the main target network, as shown in Fig. 1D. For aspirin and OA combination therapy to enhance CRC chemotherapy, these targets were considered potential candidates.

An analysis of 329 common targets by the String database was conducted to construct a PPI network using OA to promote CRC treatment with aspirin. Using PPI network confidence settings of 0.9 and hiding disconnected nodes in the network, high confidence was obtained. Figure 1B(b) shows the PPI network of target proteins from the screening, which shows that 324 targets have potential protein interactions, 1912 edges representing protein interactions, and an average degree of freedom of 11.8. In order to identify key sub-networks in the network, the CytoHubba ranking method was used, followed by the MCC topology analysis method. The top 10 key target proteins were IL6, IL10, TNF, IL1B, CXCL8, IL1A, CCL2, CSF2, CSF3, and CCL5, which were considered to be OA-enhanced core targets of aspirin therapy for CRC, and the key sub-network of OA-aspirin-CRC is shown in Fig. 1B(c).

### GO enrichment analysis

**Fig. 2:**
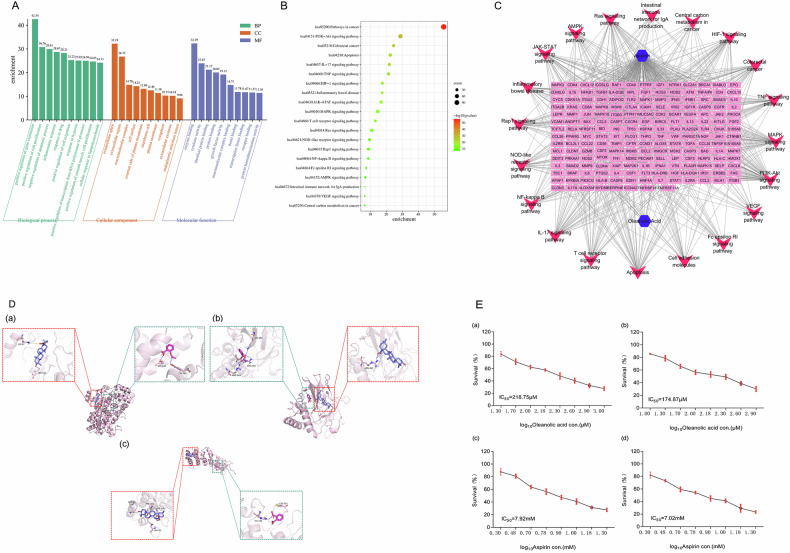
Mechanisms of oleanolic acid and aspirin in the treatment of colorectal cancer. **A** Analyzes of GO enrichment for oleanolic acid and aspirin in colon cancer; **B** Analyze of KEGG signaling pathways enriched in oleanolic acid, aspirin, and colon cancer; **C** Oleanolic acid enhances the anti-colorectal cancer effects of aspirin by modulating the activation of target pathways; **D** Diagram of molecular docking patterns (**A**: Aspirin-Oleanolic Acid-COX2 binding mode; **B**: Aspirin-Oleanolic Acid-NFκB1 binding mode; **C**: Aspirin-Oleanolic Acid-IκBα binding mode. The green stick represents oleanolic acid; the red stick represents aspirin; the white cartoon represents protein; and the yellow line represents hydrogen bonds.); **E** CCK8 assays reveal the inhibiting effects of oleanolic acid and aspirin on colorectal cancer cell proliferation ((a and b): IC_50_ of oleanolic acid and aspirin on HCT-116 and HT-29 cells were 218.75 and 174.87 μM, respectively; (c and d): IC_50_ of oleanolic acid and aspirin on HCT-116 and HT-29 cells were 7.92 and 7.02 mM, respectively).

GO functional enrichment analysis includes three components: biological process (BP), molecular function (MF), and cellular component (CC). The component-disease intersection targets were imported into the DAVID database, and the species was limited to “Homo Sapiens”, and the rest were set to default for GO enrichment analysis. We found 1206 BPs, including genes regulated positively, proliferating cells regulated positively, and apoptotic cells regulated negatively; 121 CCs were obtained, involving extracellular space, extracellular region, macromolecular complex, etc. As shown in Fig. 2A, our analysis focused on the top 10.

### KEGG enrichment analysis

The DAVID 6.8 database was used to perform KEGG enrichment analysis on 329 target genes with the purpose of identifying potential pathways of OA-enhanced aspirin against CRC (Fig. 2B). The key pathways involved are the PI3K-Akt signaling pathway, IL17 signaling pathway, CRC, TNF signaling pathway, and NF-κB signaling pathway. Figure 2C shows the genes associated with these pathways. Multiple signaling pathways were enriched in NFκB1/IκBα/PTGS2/MAPK3/PIK3CA simultaneously, suggesting that the key targets of OA and aspirin combination therapy may be NFκB1/IκBα/PTGS2/MAPK3/PIK3CA, thereby enhancing aspirin’s effectiveness against colorectal rectal cancer.

### Binding affinity of candidate targets to compounds in oleanolic acid and aspirin

To understand the affinity of aspirin and OA, we combined PTGS2 (COX-2) with NFκB1 and IκBα molecule docking. Vina 1.1.2 software was used to predict the Aspirin-OA-PTGS2, Aspirin-OA- NFκB1, and Aspirin-OA-IκBα ternary complexes, respectively, as shown in Fig. 2D.

Aspirin and OA’s binding affinity scores with PTGS2 were −6.8 and −8.8 kcal/mol, respectively. The binding affinity scores of Aspirin and OA with NFκB1 were −4.9 and −6.8 kcal/mol, respectively, and the binding affinity scores of Aspirin and OA with IκBα were −5.8 and −8.0 kcal/mol. There is evidence that Aspirin and OA could bind to PTGS2 and NFκB1 proteins simultaneously, which could contribute more to the stable formation of OA/Aspirin complexes.

It is mainly through hydrogen bonds between small molecule ligands and amino acid residues in the protein that the conformation binds strongly to large molecule proteins. Hydrophobic forces are generated in the conformation and, therefore, increase the binding affinity of the protein. A diagram illustrating the interaction between Aspirin, OA, and PTGS2 can be seen in Fig. 2D(a). The Aspirin binding site was previously reported [28] as being among the active sites of PTGS2. In contrast, OA binds on the surface of PTGS2, which is the entrance of Aspirin. Figure 2D(a) illustrates that OA forms hydrogen bonds with ASP-347 and also undergoes salt-bridge interactions with LYS-97 to enhance its binding to small molecules and proteins. On the right panel of Fig. 2D(a), we can see that Aspirin forms hydrogen bonds with SER-530 and TYR-385 on NFκB1 protein. The left panel of Fig. 2D(b) shows how Aspirin forms a hydrogen bond with ARG-484, ARG-461, and LYS-392 on PTGS2. OA can be seen in the right panel, forming a hydrogen bond interaction with ARG-461 on the PTGS2 protein. As shown in Fig. 2D(c), in the interaction diagram of the Aspirin-Oleanolic_acid-IκBα ternary complex, Aspirin binds to the Helix surface of the IκBα protein. Oleanolic_acid binds to another Helix surface of IκBα protein. From the left picture, we can see that Oleanolic_acid forms a hydrogen bond with GLN-96 on IκBα protein. In addition, hydrophobic contact with surrounding VAL-97, PHE-103, VAL-93, PHE-77, PEH-106, and LEU-70 further strengthens protein and small molecule binding. It is clear from the figure on the right that Aspirin forms hydrogen bonds with HIS-184 on the IκBα protein.

These amino acids are the main factors that maintain the protein and the two small molecules bound to each other. Both molecules are capable of interacting well with the protein based on their binding patterns. The stable binding of the small molecule Oleanolic_acid to the surface of PTGS2, NFκB1, and IκBα pockets, respectively, contributes to the more durable stabilization of Aspirin at the binding site.

### In vitro experimental validation

#### A study of oleanolic acid and aspirin’s inhibitory effects on human colorectal cancer cells

To evaluate the antitumor activity of OA and aspirin in human CRC cells, we treated HCT-116 and HT-29 CRC cells with different concentrations of OA and aspirin for 48 h. In a concentration-dependent manner, both OA and aspirin reduced CRC cell proliferation. The IC_50_ of OA and aspirin for these two CRC cell lines were 218.75 μM, 174.87 μM, 7.92 mM, and 7.02 mM, respectively (Fig. 2E).

### An oleanolic acid/aspirin combination therapy inhibits human colorectal cancer cell growth synergistically

**Fig. 3:**
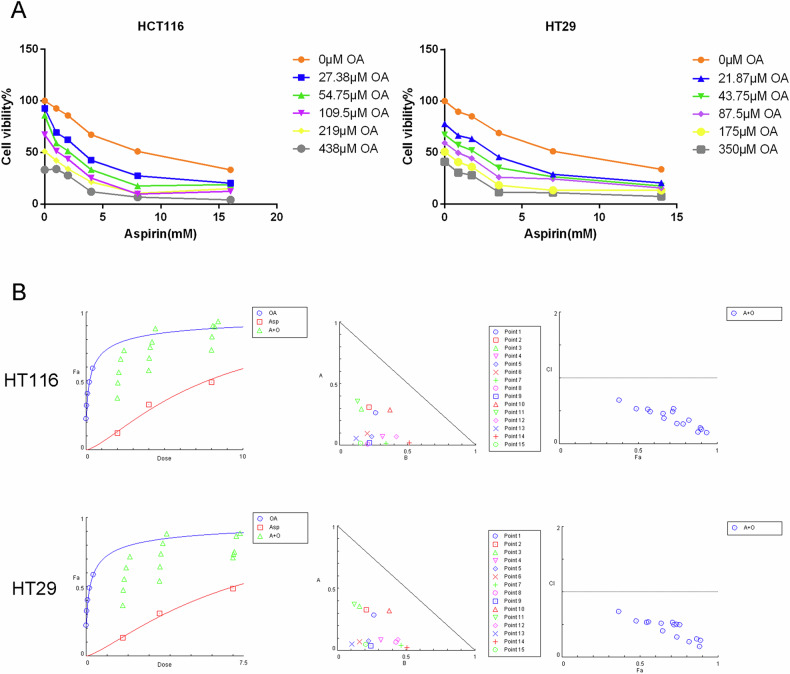
Synergistic effect of oleanolic acid in combination with aspirin. **A** After 48 h of treatment with the oleanolic acid and aspirin combination, the inhibitory effect of oleanolic acid and aspirin was observed on the proliferation of HCT-116 and HT-29 cell carcinomas. **B** Computer simulations of the Dose-Effect Curve, isobologram, and Fa-CI maps of oleanolic acid and aspirin combination in HCT-116 and HT-29 cell lines were performed using CompuSyn software.

To determine if there is a potential significance to the synergistic inhibition of CRC by OA and aspirin, we determined the inhibitory effects of OA, aspirin, and different ratios of OA/aspirin combination on human CRC HCT-116 and HT-29 cells. According to the IC_50_ values of OA and aspirin, HCT-116 and HT-29 cells were treated with OA, aspirin, and their combinations for 48 h. The results showed (Fig. 3A, B) that OA or aspirin treatment alone produced a dose-dependent growth inhibition on HCT-116 and HT-29 cells. HCT-116 and HT-29 cells were significantly inhibited by OA combined with aspirin compared to aspirin alone at corresponding concentrations.

By analyzing and calculating the combined index (CI) and related indexes of HCT-116 and HT-29 cells treated with OA combined with aspirin using Compusyn software, the synergistic anticancer effect was evaluated. Using HCT-116 and HT-29 cells treated with varying concentrations of OA and aspirin, the Dose-Effect Curve, isobologram, and Fa-CI maps were drawn. Figure 3B and Table 1 show the synergistic effect of the two combinations used in this study. The CI of all OA/aspirin dose pairs is less than 0.7, showing that OA and aspirin have a synergistic effect on the proliferation of HCT-116 and HT-29 cells in the detected concentration range. In addition, the CI values of OA/aspirin (438 μM/8 mM and 350 μM/3.5 mM) on HCT-116 and HT-29 cells were 0.17 and 0.16, respectively, indicating that the synergistic effect of OA and aspirin was the greatest at these two concentration ratios. The combination of OA and aspirin significantly inhibited the growth of human CRC cells at concentration ratios of 438 mM/8 mM and 350 mM/3.5 mM, respectively.

**Table 1:** The combination index (CI) values of combinations of oleanolic acid with aspirin in two cell lines.

HCT116		HT29	
Combinations	CI values	Combinations	CI values
Asp (2 mM) + OA (27.38 μM)	0.66	Asp (1.75 mM) + OA (21.87 μM)	0.7
Asp (2 mM) + OA (54.75 μM)	0.53	Asp (1.75 mM) + OA (43.75 μM)	0.55
Asp (2 mM) + OA (109.5 μM)	0.53	Asp (1.75 mM) + OA (87.5 μM)	0.54
Asp (2 mM) + OA (219 μM)	0.46	Asp (1.75 mM) + OA (175 μM)	0.52
Asp (2 mM) + OA (438 μM)	0.49	Asp (1.75 mM) + OA (350 μM)	0.49
Asp (4 mM) + OA (27.38 μM)	0.49	Asp (3.5 mM) + OA (21.87 μM)	0.53
Asp (4 mM) + OA (54.75 μM)	0.39	Asp (3.5 mM) + OA (43.75 μM)	0.4
Asp (4 mM) + OA (109.5 μM)	0.31	Asp (3.5 mM) + OA (87.5 μM)	0.31
Asp (4 mM) + OA (219 μM)	0.30	Asp (3.5 mM) + OA (175 μM)	0.23
Asp (4 mM) + OA (438 μM)	0.18	Asp (3.5 mM) + OA (350 μM)	0.16
Asp (8 mM) + OA (27.38 μM)	0.53	Asp (7 mM) + OA (21.87 μM)	0.53
Asp (8 mM) + OA (54.75 μM)	0.36	Asp (7 mM) + OA (43.75 μM)	0.50
Asp (8 mM) + OA (109.5 μM)	0.22	Asp (7 mM) + OA (87.5 μM)	0.49
Asp (8 mM) + OA (219 μM)	0.24	Asp (7 mM) + OA (175 μM)	0.28
Asp (8 mM) + OA (438 μM)	0.17	Asp (7 mM) + OA (350 μM)	0.25

In addition to the CI value compared to 1, a more refined CI division is as follows: strong synergy CI = 0.1–0.3, synergy CI = 0.3–0.7, medium synergy CI = 0.7–0.85, slight synergy CI = 0.85–0.9.

According to the inhibition rate of different concentrations of OA, aspirin, and their composite drugs on two kinds of cells and IC_50_ calculations, the following principles were set to set the concentration of the administered drugs: Within the range of synergistic effect, the effective single drug concentration lower than the IC_50_ concentration was higher than the compound drug concentration of IC_50_, while the high inhibition rate group retained a certain cell survival rate, that is, 20 and 50 μM of OA and aspirin 4 mM. Therefore, the concentration was selected for the follow-up experiment.

### Oleanolic acid enhances the effect of aspirin on the proliferation and invasive ability of colorectal cancer HCT-116 and HT-29 cells

**Fig. 4:**
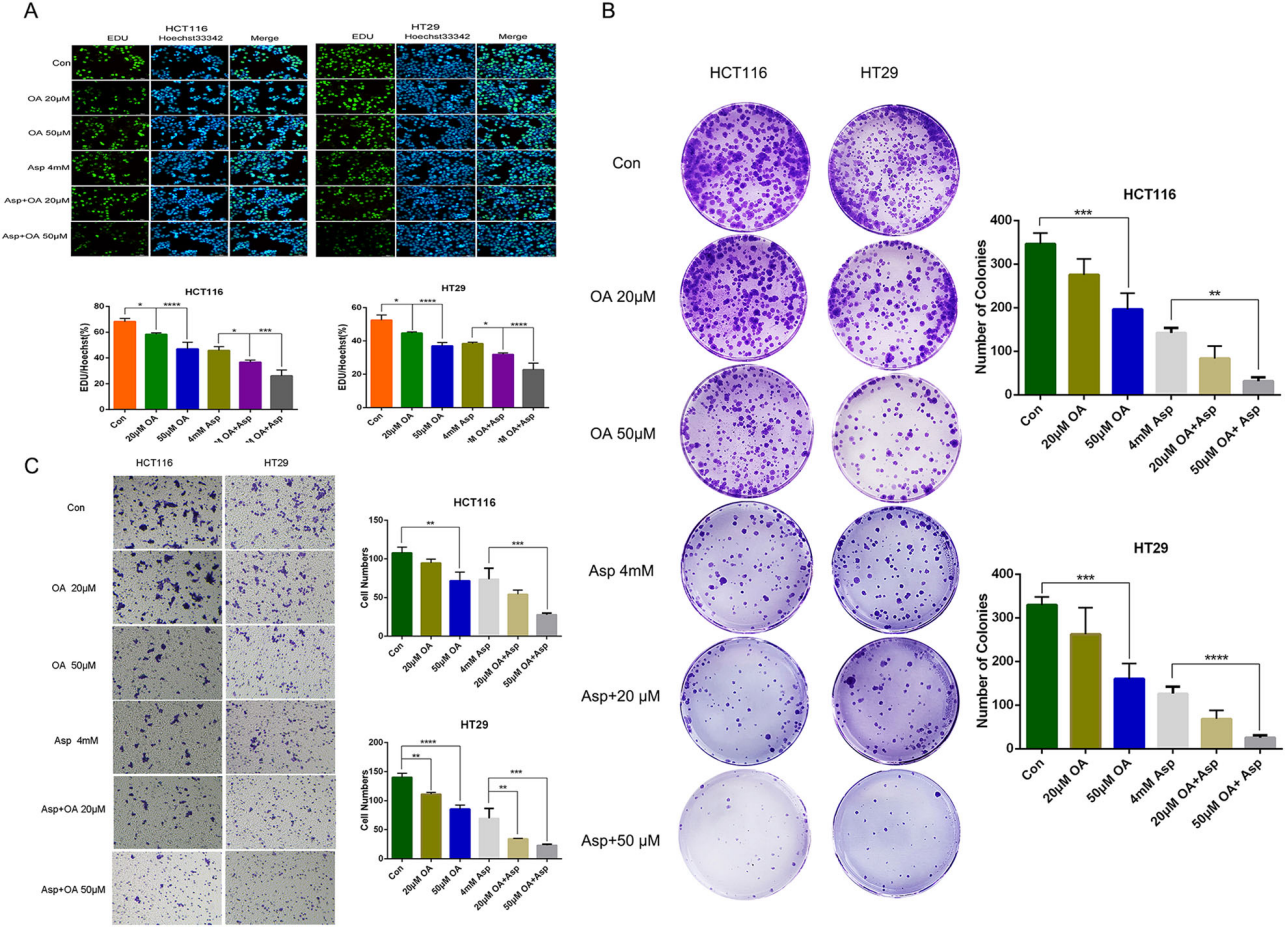
Effects of oleanolic acid and aspirin and their combination on the proliferation and invasive ability of colorectal cancer HCT-116 and HT-29 cells. **A** Proliferation inhibition by oleanolic acid and aspirin detected by EDU labeling of HCT116 and HT29 cells. (Blue fluorescence represents cells labeled with Hoechst 33342, and green fluorescence represents proliferating cells undergoing DNA replication labeled with EdU. Fluorescence signals were quantified with Image J software and displayed as mean ± SD (*n* = 3) in the form of bar graphs). **B** Colony formation assay to detect the inhibitory effect of oleanolic acid and aspirin on the 48 h proliferation of HCT116 and HT29 cells. **C** Transwell assay to detect the inhibitory effect of oleanolic acid and aspirin on the 48 h invasive ability of HCT116 and HT29 cells. *P < 0.05, ***P < 0.001, ****P < 0.0001.

EdU experiment was conducted to detect the ability of cells to synthesize DNA in order to determine if OA and aspirin had synergistic effects on colon cancer cell proliferation. Under a fluorescence microscope, the EdU-positive nuclei (AlexaFluor488 azide labeled green) and all Hoechst-stained nuclei (blue) were observed. The proportion of proliferating cells was reflected by the statistical ratio of EdU-positive nuclei to Hoechst-stained nuclei. Results showed that (Fig. 4A) OA and aspirin reduced the proportion of proliferating cells (*P* < 0.05), and this proportion was also significantly reduced in the synergistic dosing group of OA and aspirin compared to the Asp monotherapy group (*P* < 0.05), further confirming the synergistic inhibition of cellular proliferation by the combination of OA and aspirin. In the meantime, the results of colony formation assay (Fig. 4B) and invasion assay (Fig. 4C) further confirmed that OA was able to inhibit the proliferation and invasion ability of CRC cells and had a synergistic enhancement effect in combination with Asp.

### Oleanolic acid/aspirin synergistically induces apoptosis in colorectal cancer cells

**Fig. 5:**
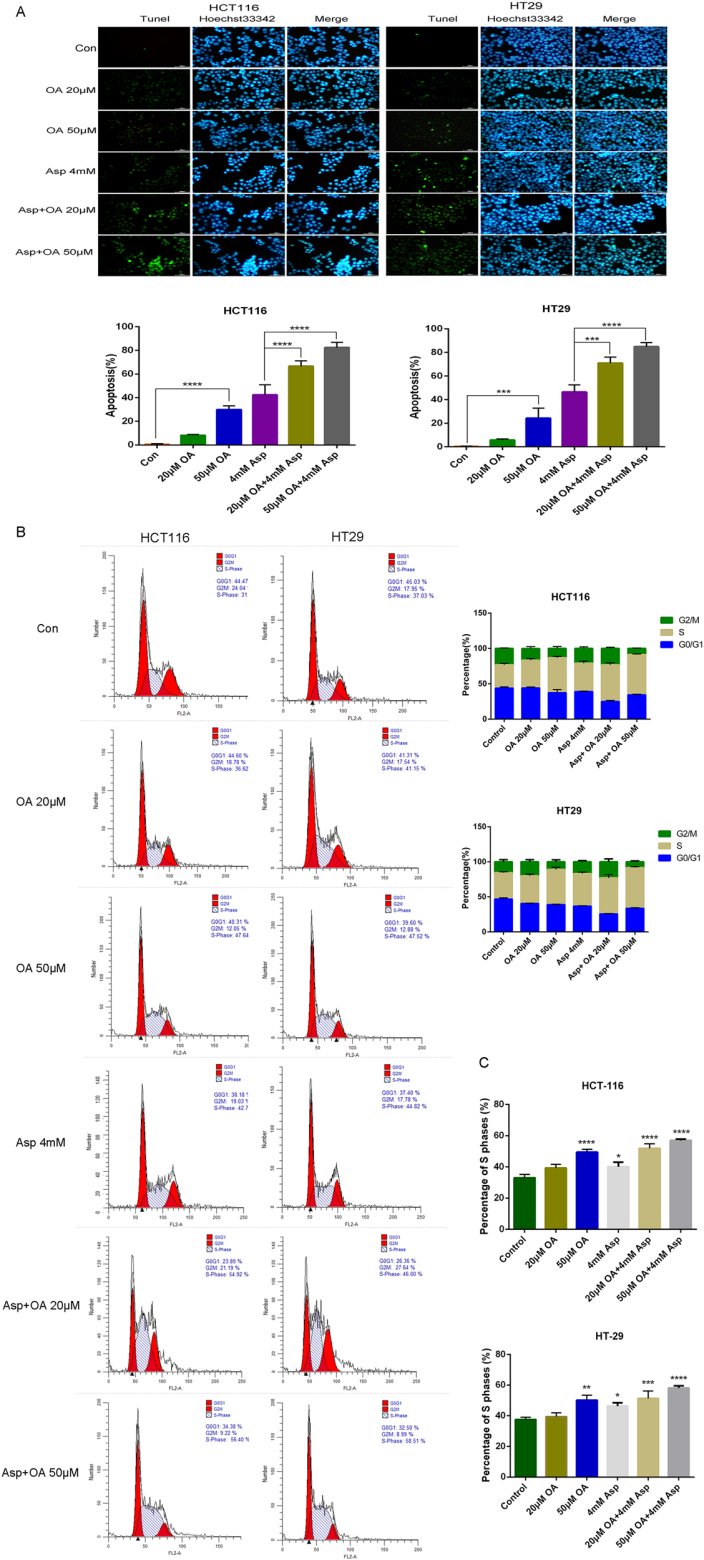
Effects of oleanolic acid, aspirin and their combination on apoptosis-inducing effects and cell cycle progression in HCT116 and HT29 cells. **A** Apoptosis-inducing effects of oleanolic acid, aspirin, and their combination on HCT116 and HT29 cells detected by Tunel assay. (Tunel results are presented as bar graphs as mean ± SD (*n* = 3)). ***P < 0.001, ****P < 0.0001. **B** Effects of OA, Asp and their combination on cell cycle progression in HCT116 and HT29 analyzed by flow cytometry. **C** Bar chart of percentage of cells in phase S.

During apoptosis, genomic DNA breaks and exposed 3′-OH can be catalyzed by TdT plus FITC-labeled dUTP, as measured by TDT-mediated dUTP end labeling (TUNEL). In HCT116 cells, the number of apoptotic cells was significantly higher in the OA group than in the control group (Fig. 5A), and the apoptosis rate increased as a function of concentration gradient. There was a statistically significant difference between the high-concentration group and the control group. There was a significant difference in apoptosis rates between the Asp and control groups (*P* < 0.0001). The apoptosis rate was increased in the Asp group compared to that in the apoptosis rate increased in the combined drug group (*P* < 0.001), while similar results were presented in HT29 cells. This study shows that colon cancer cells had significant apoptosis after being treated with OA and aspirin.

### Cell cycle analysis

The cell cycle distribution of HCT116 and HT29 cells was detected by flow cytometry, and the results showed (Fig. 5B, C) that in HCT116 cells, compared with Control group, S-phase cells were increased in the OA group in a concentration gradient-dependent manner (*P* < 0.0001 in the high-concentration group), and S-phase cells were increased in the Asp group (*P* < 0.05); compared with the Asp group, the S-phase in the co-administration group cells increased (*P* < 0.001). Similar results were presented in HT29 cells, which, combined with the cell inhibitory proliferative effects of OA and Asp and the combined application, indicated that OA and Asp and the combined drug treatment affected the cell cycle regulation and caused the cells to undergo S-phase arrest; the results indicated that OA could induce S-phase arrest in HCT116 and HT29 cells alone, and that the combination with Asp had a synergistic effect on the cell cycle process in combination with Asp has a synergistic regulatory effect. An increase in the S phase of the flow cycle may be due to vigorous cell proliferation, increasing proliferating cells. Another possibility is that due to the inhibition of cell proliferation, DNA synthesis requires more time or induces DNA damage, and a malfunction occurs in the later stages of DNA synthesis, namely S-phase arrest. Combined with CCK8, EDU, and Colony formation assay, it was found that cell proliferation was inhibited.

### OA/Asp synergizes to induce ROS-mediated cell damage in colorectal cancer cells synergistically

**Fig. 6:**
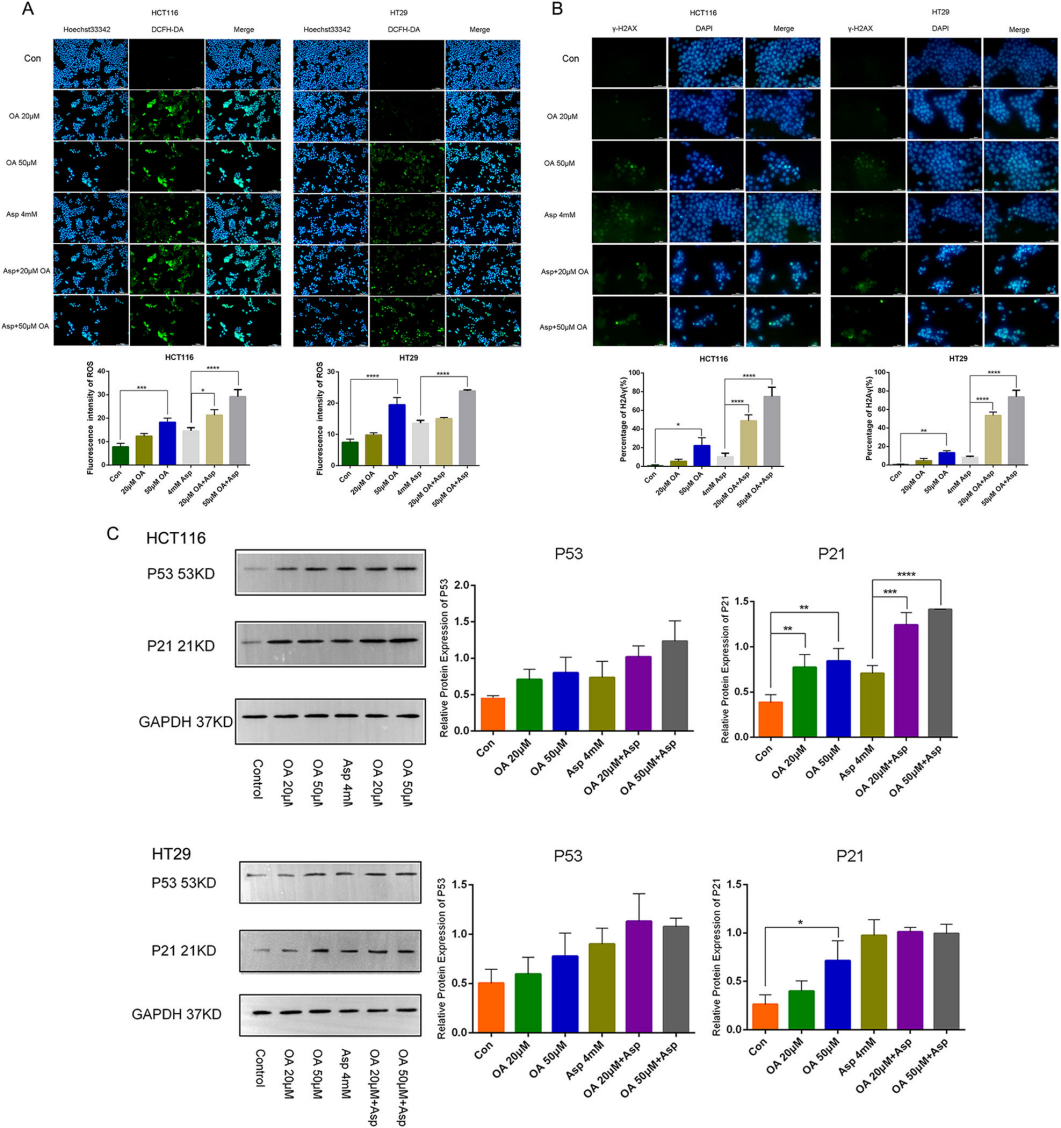
OA/Asp synergizes to induce ROS-mediated cell damage in colorectal cancer cells synergistically. **A** Detection of intracellular ROS production and quantitative analysis of ROS expression based on DCF fluorescence intensity using DCFH-DA. **B** Determination of DNA damage by immunofluorescence. **C** Western blot assay to detect the effects of OA, Asp and their combined treatment on P53 and P21 protein expression in colon cancer cells.

To determine whether OA, Asp and their combined treatments induced ROS-mediated cellular damage in colon cancer cells, intracellular ROS levels and the DNA damage marker γH2A.X were detected by staining using the DCFDA probe and immunofluorescence assay according to the manufacturer’s instructions after OA and Asp treatment. As shown in Fig. 6A, the results indicated that the fluorescence intensity of ROS in colon cancer cells was significantly increased after 48 h of OA and Asp treatment compared with the control group. Immunofluorescence staining analysis showed that the proportion of positive cells for γH2A.X in colon cancer cells increased after OA, Asp treatment. The expression was significantly enhanced in the combination group (Fig. 6B). It indicated that OA and Asp promoted cell damage and death by inducing ROS production and oxidative DNA damage, and the combination of OA and Asp had a more significant and synergistic effect.

It has been shown that when DNA damage occurs, the accumulation of DNA damage triggers the DNA damage response mechanism to repair the damaged DNA and cell cycle arrest occurs, thus providing sufficient time for DNA repair. p53 is a key factor in the intrinsic cellular response to DNA damage. Activation of p53 by phosphorylation of serine-15 leads to the upregulation of p21, a target gene downstream of p53, which plays a role in repairing damaged DNA and blocking the cell cycle. It has also been suggested that p21 expression may exert its role in blocking cellular processes and preventing replication of damaged DNA through a form that is not dependent on p53 activation [29, 30]. The results of protein western blotting of p53 and p21 showed that the relative expression of p53 and p21 proteins in colon cancer cells treated with OA, Asp and their combination was up-regulated compared with the control group, suggesting that p53-mediated or unmediated cell cycle arrest and DNA repair processes occurred in cells treated with OA, Asp and OA + Asp. When colon cancer cells were treated with OA, Asp and OA + Asp, a large number of cells were arrested in the S phase of the cell cycle, associated with increased expression of p53 and p21 proteins (Fig. 6C), which suggests that DNA damage, cell cycle checkpoint proteins were activated and consequently induced the cell cycle to undergo S-phase arrest. The original diagram of the western blot assay in Fig. 6C is shown in Supplementary Figs. S3 and S4 in the Supplementary Material.

In conclusion, the effects of OA and Asp inhibited cell proliferation and promoted apoptosis, and the combination of OA and Asp had a synergistic effect compared with that of the single agent group. OA, Asp, and their combination treatment increased intracellular ROS, leading to oxidative DNA damage, activation of DNA repair, and blockage of the cell cycle.

### Effects of oleanolic acid, aspirin, and their combination on core target genes in colorectal cancer HCT116 and HT29 cells

**Fig. 7:**
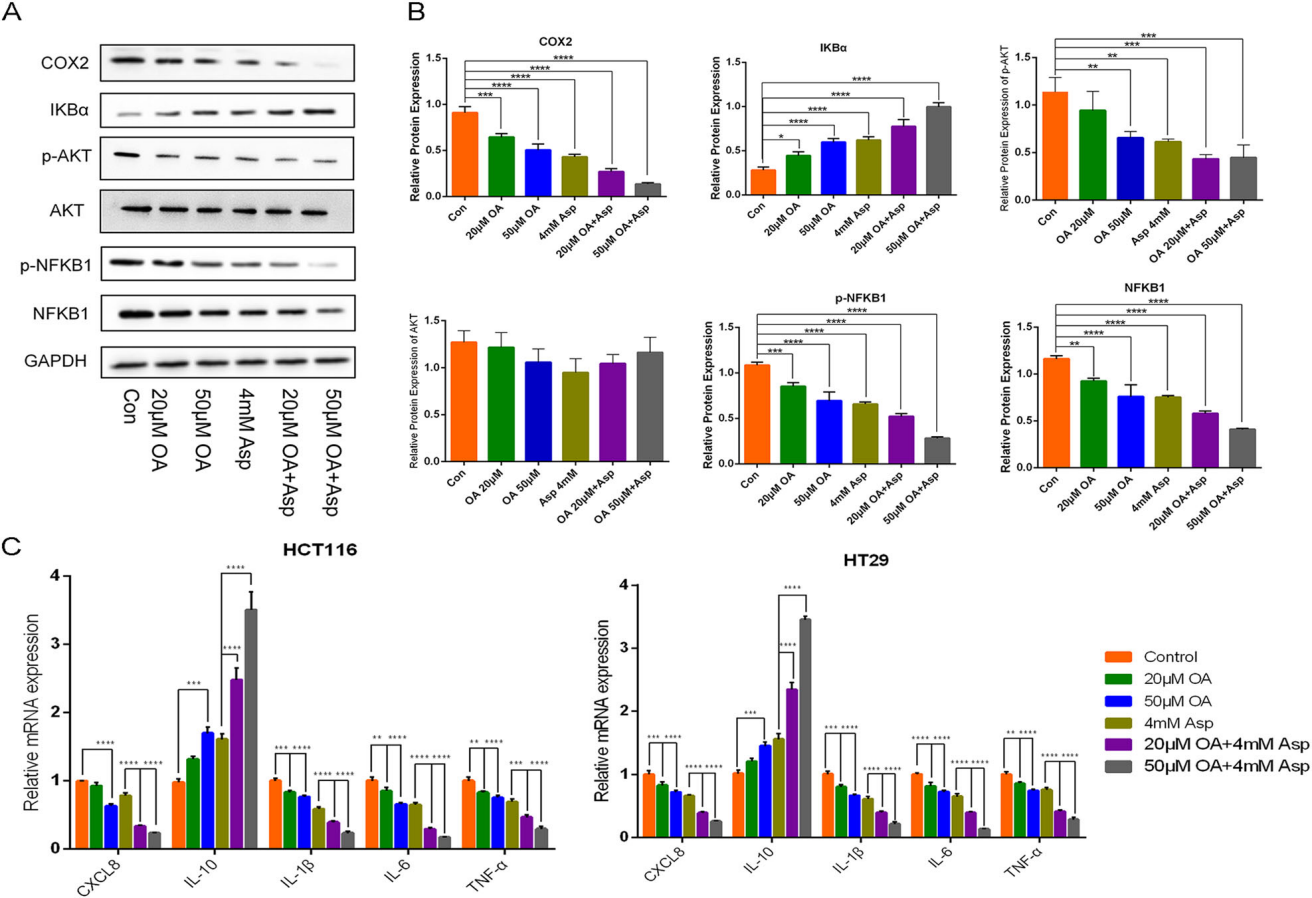
The combined application of oleanolic acid and aspirin to inhibit colorectal cancer cell growth was examined by RT-qPCR and western blotting. **A**, **B** Effects of oleanolic acid, aspirin, and their combination on Akt/NF-B/IBα/COX2 signaling pathway in colorectal cancer HCT116 and HT29 cells detected by western blot assay. **C** Effect of oleanolic acid, aspirin, and their combination on core target genes in colorectal cancer HCT116 and HT29 cells by RT-qPCR assay.

RT-qPCR analysis of the core target genes ranked among the top 5 in terms of degrees of freedom in the PPI protein-interacting network was performed to understand further the effects of OA, aspirin, and their combined application on HCT116 and HT29 cells. The RT-qPCR results (Fig. 7C) showed that OA, aspirin, and their combined application elevated the expression of IL10 and decreased IL6, TNF-α, IL1B, and CXCL8 mRNA transcript levels.

### Oleanolic acid, aspirin, and their combination inhibit colorectal cancer cell growth by modulating Akt/NF-κB/IκBα/COX2 signaling pathway

HCT-116 and HT-29 cells were treated with OA and aspirin to examine their mechanism of action on growth inhibition. Western blotting was used for the subsequent study. To date, a number of studies have confirmed that the PI3K/AKT pathway is able to cross-react with the NF-κB pathway and regulate its activity to some extent [31, 32]. The PI3K-AKT and NF-κB signaling pathways are involved in a number of important pathways in the organism, including metabolism, angiogenesis, cell cycle, apoptosis, cell proliferation, and inflammatory response. PI3K can modify the protein structure of Akt and specifically activate it after binding to signals such as growth factor receptors: PI3K receives extracellular signals, phosphorylates PIP2 to PIP3, and the phosphoryl group at position 3 of PIP3 can recruit PDK1 and AKT simultaneously so that the regulatory gene PDK1 phosphorylates the threonine at position 308 of the AKT protein (T308), leading to partial activation of AKT; further phosphorylation of AKT serine 473 sites after the completion of AKT activation, the protein structure of Akt and its activation [33, 34]. Activation of AKT links inflammation, and NF-κB would have been inactivated by binding to the inhibitory protein IκB to form a trimeric complex, and activated AKT is able to activate IKKs, leading to phosphorylation and degradation of IκBα, which results in the release of NF-κB and activation of the NF-κB signaling pathway. In patients with colitis, the binding of inflammatory factors to the receptor by intestinal mucosal tissue cells activates IκB kinase through the upstream PI3K/AKT signaling pathway, prompting serine phosphorylation of the phosphorylation site of the NF-κB·IκB complex, which in turn leads to ubiquitinyl modification and promotes the release of large quantities of NF-κB in patients [35, 36]. NF-κB downstream regulates COX-2 transcription, leading to increased COX-2 expression, which promotes the production of a variety of inflammatory transmitters and amplifies and sustains inflammation [37]. Clinical studies have confirmed that inhibition of this pathway can suppress CRC proliferation [38], consistent with our results (Fig. 7A, B). The original diagram of the western blot assay in Fig. 7A, B is shown in Supplementary Fig. S5 in the Supplementary Material. The degree of phosphorylated AKT was reduced by OA, Asp and their combined treatment, and in the results of HCT116 cells, the difference between 50 μM of the OA group, Asp group and OA + Asp group in the combination group compared with the control group was significant (*P* < 0.01). The expression of NFκB1 was downregulated; the difference was significant (*P* < 0.01) compared with the control group; the expression of COX2 protein was downregulated in the OA group, Asp group and combination group, and the difference was significant (*P* < 0.001). The experimental results indicated that the phosphorylation of AKT and NFκB1 was inhibited, while the expression of COX2 protein was decreased in the cells treated with OA, Asp and their combination, and the mechanism may be through the inhibition of the activation of PI3K/AKT signaling pathway and NF-κB signaling pathway; and at the same time downregulation of the expression of COX2 protein, which can inhibit the proliferation and survival of cancer cells.

## Discussion

The worldwide death rate from malignancy is one of the leading causes of death, and CRC is one of the most common and fatal causes of death, accounting for more than 900,000 deaths by the year 2020 [1]. Long-term chemotherapy regimens using current chemotherapeutic agents are not sufficiently effective, and adverse effects can vary greatly [39, 40]. The cause of the vast majority of CRCs can be attributed to dietary factors, and recent studies have shown that a variety of natural compounds isolated from fruits, vegetables, and medicinal plants have CRC-preventative effects. They can effectively enhance the chemotherapeutic effect of chemotherapeutic agents by Combining conventional chemotherapy with other treatments [41]. A growing number of studies have shown that the combination of natural products with conventional chemotherapeutic agents can achieve more favorable outcomes, such as enhanced efficacy, dose reduction, and reduced or delayed development of drug resistance [40, 42, 43]. Understanding the left-right mechanism between these compounds may assist in guiding their clinical application. Combination therapies are widely used in the clinical treatment of cancer.

There is increasing evidence that triterpenoids may have physiological effects, including those related to hepatoprotection, analgesia, anticancer, anti-inflammatory, and immunomodulation. It is through the breakdown of triterpene saponins in the intestine that triterpenoids are released, which are absorbed and integrated into cell membranes to regulate various genes [44]. OA, pentacyclic triterpenic acid, is a triterpene acid isolated from a variety of medicinal plants. OA has been previously studied for its anticancer potential in vitro and in vivo. The mechanism by which it inhibits colon cancer is still in the process of being elucidated. The development of more precise and targeted therapeutic approaches may be enabled by understanding the molecular actions of these compounds. OA is relatively non-toxic and cytotoxic to many types of cancer cells. In addition to targeting tumor cells by inducing apoptosis, it can also modulate the tumor microenvironment, exhibiting anti-angiogenesis and anti-cellular differentiation [45]. OA is a potentially effective antitumor agent against a variety of tumors, including CRC.

Inflammation-induced colorectal carcinogenesis can be inhibited by aspirin by reducing COX2 and ROS levels, thus preventing DNA damage and YAP1 oncogenicity. Aspirin has been shown to inhibit inflammation-induced colon cancer by reducing COX2 and ROS levels [46]. Researchers have demonstrated that aspirin combined with natural compounds can enhance its anticancer effects. Zhu et al. constructed a resveratrol-based aspirin derivative as a prodrug (RAH) that released both resveratrol and aspirin in vivo, which could enhance the anticancer and anti-inflammatory activities of aspirin while reducing gastric toxicity. RAH inhibits cyclin in mice by downregulating cell cycle arrest and induces apoptosis in cancer cells through activation of caspase-3 [47]. It is therefore hypothesized that the combination of OA and aspirin could improve the effects of aspirin on CRC, as well as to provide a theoretical basis for the preparation of aspirin derivatives derived from OA, as well as to provide an idea for developing new drugs for CRC treatment.

An examination of aspirin’s and OA’s potential mechanisms against CRC was conducted using network pharmacology. We obtained 78 OAs, 978 aspirins, and 1558 target genes associated with CRC. An interconnection network between disease targets and component targets was constructed by intersecting 329 common targets. The PPI results showed that the target genes of IL6, IL10, TNF, IL1B, CXCL8, IL1A, CCL2, CSF2, CSF3, and CCL5 might be the core targets of OA-enhanced aspirin therapy for CRC. Multiple signaling pathways were enriched in NFκB1/IκBα/PTGS2/MAPK3/PIK3CA simultaneously, suggesting that the key targets of OA and aspirin combination therapy may be NFκB1/IκBα/PTGS2/MAPK3/PIK3CA, thereby enhancing aspirin’s effectiveness against colorectal rectal cancer. Molecular docking revealed that Aspirin-OA docked with both PTGS2, NFκB1, and IκBα to form Aspirin-OA-PTGS2, Aspirin-OA-NFκB1, and Aspirin-Oleanolic_acid-IκBα Binding affinity for both ternary complexes.

The purpose of this study was to identify the mechanism of how OA and aspirin complement each other to enhance aspirin’s anti-CRC effects. The CRC cell lines HT-29 and HCT-116 were selected for the study. The synergistic proliferation inhibitory effects of OA and aspirin were determined by CCK8 assay and isobologram analysis. EdU cell proliferation assay was used to detect the effect of the combination on the DNA synthesizing ability of HCT116 and HT19 cells. The apoptosis and cell cycle of HCT116 and HT19 cells were detected by the Tunel method and flow cytometry. On HCT-116 and HT-29 cells, RT-qPCR and western blotting were used to determine the molecular mechanisms involved with the combined drug. We show that OA synergistically enhanced aspirin’s apoptotic effects in HCT116 and HT29 cells by regulating the Akt/NFκB/IκBα/COX2 signaling pathway and inducing cellular S-phase arrest.

Isobologram maps have been widely used to evaluate drug interactions and to detect whether enhanced proliferation inhibition is synergistic [48]. On HCT116 and HT29 cells, OA, aspirin, and their combination were measured, and the IC value was calculated, revealing that both inhibit CRC cell proliferation dose-dependently. Likewise, aspirin alone had a much stronger inhibitory effect on HCT-116 and HT-29 cells than OA in combination with aspirin. EdU cell proliferation assay was used in order to detect the effect of the combination of drugs on the DNA synthesis ability of HCT116 and HT19 cells. Cell cloning and cell invasion assays were used to validate further the effects of OA and Asp and their combination on the proliferation and invasion of HCT116 and HT19 cells, and the effects of the combination of drugs on apoptosis in HCT116 and HT19 cells were detected by the Tunel method and flow cytometry and cell cycle. Detection of intracellular ROS and DNA damage marker γH2A.X levels confirmed the synergistic induction of ROS-mediated cellular damage by OA and Asp and their combined application in CRC cells. Moreover, the results of protein immunoblotting of p53 and p21 showed that p53-mediated or unmediated cell cycle arrest and DNA repair processes occurred in cells treated with OA, Asp, and OA + Asp. When colon cancer cells were treated with OA, Asp and OA + Asp, a large number of cells accumulated in the S-phase of the cell cycle, and S-phase arrest was correlated with an increase in the expression of p53 and p21 proteins, suggesting that DNA damage activation of cell cycle checkpoint proteins and consequently induced S-phase arrest of the cell cycle occurred.

The expression of COX-2 is increased in patients with CRC and is associated with a lower survival rate and poor prognosis [49]. In the context of CRC, aspirin has proven to be a highly effective chemopreventive agent, primarily through direct inhibition of COX-2. Almost all CRCs with COX-2 positivity appear to benefit from taking aspirin to reduce cancer risk [50]. Studies have shown that regular aspirin use is associated with a significant reduction in CRC incidence. Not only that, in some CRCs with COX-2 and PIK3CA mutations, regular aspirin use was associated with better prognosis and clinical outcomes [51–53]. Aspirin may inhibit cancer cell proliferation and survival by inhibiting COX-2 expression and reducing prostaglandin (PG) E2 synthesis, thereby reducing the inflammatory response [51, 53]. The nuclear transcription factor NF-*κ*B is particularly important in the development of CRC. Aberrant regulation of this signaling pathway was observed in about 60–80% of CRC cases [54]. NF-κB is a dimer that exists in the cytoplasm in an inactivated state in the absence of stimulation by binding NF-κB and its corresponding repressor protein IκB. IκB is an inhibitory protein of NF-κB and a component of the activation pathway of NF-κB, of which IκBα is one of the important members of the IκB family, as it can most rapidly phosphorylate. It becomes a key component in the inhibition of NF-κB activity [55].

Studies have shown that aspirin derivatives inhibit the NF-κB signal pathway by inhibiting the degradation of IκB molecules [56]. The activation of AKT is associated with the occurrence of inflammation. Activated AKT can activate IKKs, leading to the phosphorylation and degradation of IκBα, thus activating the NF-κB signal pathway. Huang Qi et al. [57] showed that the expression of AKT was correlated with tumor differentiation, lymph node metastasis, distant metastasis and TNM stage. AKT is an independent factor affecting the prognosis of patients with CRC. Aladhraei et al. [58] found that NF-κB expression may be a potential biomarker for the onset and proliferation of CRC. Phosphorylated AKT separates from the cell membrane, activates IKK, and leads to NF-κB inhibits protein dissociation. Therefore, NF-κB is released from the cytoplasm and transferred to the nucleus, which can activate different target genes and produce effects. Perifosine is a new orally targeted AKT inhibitor, which is still in phase III clinical research and development, mainly for the treatment of patients with CRC and multiple myeloma [38], consistent with our results (Fig. 7A, B). OA and Asp may inhibit the activation of the NF-κB signaling pathway by inhibiting the expression of AKT and up-regulating the expression of IκBα protein while downregulating the expression of COX2 protein and inhibiting the synthesis of inflammatory transmitters such as prostaglandin (PG) E2, thus reducing the inflammatory reaction and inhibiting the proliferation and survival of cancer cells. At the same time, the combined application of OA and Asp shows a synergistic effect. These results suggest that the combination of OA and Asp may activate the Akt/NFκB/IκBα/COX2 signal pathway in the proliferation and apoptosis of CRC cells.

In conclusion, this study explored the possible mechanism of treatment for CRC by combining OA and aspirin using network pharmacology and molecular docking techniques. We found that the combination of OA and aspirin may act on CRC through multi-target and multi-pathway effects to inhibit tumorigenesis and progression. It was verified by in vitro experiments that the combination of OA and aspirin synergistically inhibited the proliferation and invasion-promoted apoptosis and S-phase arrest of CRC cells. The mechanism of apoptosis may be the increase of intracellular ROS, leading to oxidative DNA damage and activation of DNA repair and cell cycle arrest. The molecular mechanism may inhibit the proliferation and survival of cancer cells by inhibiting the activation of the PI3K/AKT signaling pathway and the NFκB signaling pathway and simultaneously downregulating the expression of COX2 protein. The combination of OA and aspirin suggests a new therapeutic strategy for CRC. The study provides a theoretical basis for the preparation of aspirin derivatives derived from OA, but further in vivo and in vitro tests are required to validate the combination.

## Materials and methods

### Network pharmacology and molecular docking

#### An analysis of potential target genes for oleanolic acid and aspirin

Download the SDF structure of the active ingredients in PubChem (https://pubchem.ncbi.nlm.nih.gov) database. Import the SDF file into the Swiss Target Prediction database (http://www.swisstargetprediction.ch/) and Genecards database (prediction of drug targets in https://www.genecards.org/). A probability greater than 0 was included in the screening scope, and all active component targets were identified. We selected all the target genes in the study that were involved in OA and aspirin as potential targets.

### Collection of disease target genes

For genes related to CRC, search the Gene Cards (www.genecards.org) and OMIM (www.omim.org) databases with the terms “colon cancer” and “CRC” as search terms. To screen for overlapping targets, the targets of OA and aspirin, as well as CRC, were input into the bioinformatics database (http://bioinformatics.cn) and then intersected with CRC targets.

### Protein-protein interaction (PPI) network analysis

Utilizing the String 11.5 platform (https://string-db.org/cgi/) to construct the protein-protein interaction (PPI) network of OA, aspirin, and CRCintersections. For the protein interaction information, we selected Multiple proteins and set the protein species to “Homo sapients.” Imported the obtained data into the Cytoscape 3.2.1 software, generated PPI network diagrams, analyzed them by cytoHubba, and constructed the key sub-networks of overlapping targets.

### Construction of drug-disease-target network

By using Cytoscape 3.2.1, the drug-disease target network of OA, aspirin, and CRC was created from the overlapped targets. Through the cytoHubba plug-in, according to the ranking of the degree of freedom (degree) of nodes in the network, the target genes of the first 100 degrees of freedom are selected to construct the main target network.

### An analysis of the enrichment of GO and KEGG pathways

DAVID 6.8 database (https://david.ncifcrf.gov) was used to analyze GO enrichment and KEGG pathway annotations on the overlapping targets of OA, aspirin, and CRC. A plot was created using the “ggplot2” package in Rstudio.

### Molecular docking

A molecular docking study of PTGS2 (COX-2) NFκB1 and IκBα with both OA and aspirin simultaneously was conducted to further investigate the mechanism of OA and aspirin combination therapy in enhancing aspirin’s effect against CRC. The crystal structures of COX-2 (PTGS2), NFκB1, and IκBα proteins were obtained from the PDB database with PDB IDs 5IKQ [59], 3GUT [60], and 1NFI [61]. A three-dimensional model of Aspirin and OA was derived from the PubChem database by minimizing energy under the MMFF94 force field.

In this study, molecular docking work was performed using AutoDock Vina 1.1.2 software. Aspirin was first docked with COX-2 (PTGS2), NFkB1, and IκBα proteins to obtain the complexes and then docked with OA separately to obtain the ternary complexes. We chose the docked conformation of the docking output that scored highest as the binding conformation and used PyMol 2.5 to visualize and analyze the docked ternary complex.

### In vitro experiments

#### Materials and Chemicals

OA (purity, ≥98%; Lot: AF9071301) was purchased from Chengdu Alfa Biotechnology Co., Ltd. (Chengdu, China); Aspirin purchased from Jiangsu Pingguang Pharmaceutical Co., Ltd. (Xuzhou, China). The CRC cell lines HT-29 and HCT-116 were purchased from Wuxi Xinrun Biological Co., Ltd (Wuxi, China).

### Cell culture

HT-29 and HCT-116 cells were cultured in incubators at 37 °C, 5% CO_2_, DMEM high-glucose medium with 10% fetal bovine serum (GIBCO, USA; 10027-106), 2 mM l-glutamic acid, non-essential amino acids, and sodium pyruvate. HCT-116 and HT-29 cell identification letters are available in Supplementary Material S1 and S2.

### Cell viability assay

A CCK8 assay was used to determine whether OA, aspirin, or their combination inhibited human CRC cell proliferation. Logarithmic time-phase HCT-116 and HT-29 CRC cells were diluted in DMEM with 10% fetal bovine serum and inoculated into 96-well plates (Nest, China, 702001) at 5000 cells per well, 100 μl per well, and incubated for 24 h at 37 °C.

The cells were then incubated with different concentrations of (0.1, 0.2, 0.5, 1, 5, 10, 20, 50, 100, 150, 200, 400, 800, and 1000 μM) OA after adhering to the wall, different concentrations of aspirin (0.01, 0.02, 0.05, 0.1, 0.5, 1, 5, 2, 3, 4, 5, 6, 8, 10, 15, and 20 mM), and the combination of OA and aspirin, and the untreated cells served as a negative control. The wells without cells were used as blank wells. After 48 h incubation, 10 μl of CCK-8 solution (Beyotime, China, C0039) was added to each well and incubated at 37 °C with 5% CO_2_ for 1 h. Following that, the enzyme labeling instrument was used to measure the absorbance (A value) at 450 nm, and GraphPad PrismVersion was used to estimate half maximum inhibitory concentrations (IC_50_). Triplicates of each assay were performed.

### Determination of synergism by isobologram analysis

To evaluate whether OA/aspirin has a synergistic anticancer effect, a quantitative evaluation was performed using the contour and the combination index (CI). CI = (D)1/(DX)1 + (D)2/(DX)2, D is the single dose and DX is the combination dose.CI < 1, =1, and >1 indicate synergistic, additive, and antagonistic effects, respectively. Compusyn Software uses a mathematical model proposed by Chou Talalay to analyze the synergistic effects of aspirin and OA, using the median effect equation proposed by him [62].

### EdU assay

The digested cells of HCT116 and HT29 were adjusted to 5 × 10^4^/ml, and inoculated in 24-well plates, respectively. After 24 h, the drug-containing medium containing the corresponding final concentration of single and compound drugs was replaced by groups, and the incubation was continued for 48 h. Twenty micrograms of EdU working solution were added to the medium 1:1 after the cells had been treated, resulting in a final concentration of 10 micrograms EdU (Beyotime, China, C0071S). Incubation with 0.3% Triton X-100 PBS permeate for 10–15 min followed by washing with PBS and fixation with 4% paraformaldehyde; Prepare the Click Additive Solution 2 ml system: Click Reaction Buffer: 1.72 ml, CuSO_4_: 80 μl, Click Additive Solution: 200 μl, Azide 488: 4 μl; each well was incubated with 0.5 ml of Click Reaction Buffer for 30 min at room temperature and protected from light; nuclei were stained with Hoechst 33342; after the treatment was completed, each group of images was captured and analyzed by inverted fluorescence microscope.

### Colony formation assay

Cells in logarithmic growth phase were prepared into single-cell suspension containing 10% fetal bovine serum, inoculated into 6-well plates at a density of 1000 cells/well, and cultured for 24 h at 37 °C with 5% CO_2_; after the cells were grown to adherence to the wall, the drug-containing medium containing the corresponding final concentration of single and combination drugs was replaced, and the untreated cells served as a negative control group and were treated for 48 h. After treatment, the cells were replaced with complete and continued to be cultured until the cell number of most clones in the control group was more than 50; the cells were fixed with methanol for 20 min, stained with crystal violet for 20–30 min, and then photographed and counted after the excess dye was washed with PBS.

### Cell invasion assay

The effects of OA and Asp and their combination on the invasive ability of HCT116 and HT29 cells were determined using the transwell method (Costar, 3422). Matrigel was placed at 4 °C overnight to melt, and all related consumables were placed at −20 °C pre-cooled and set aside; Matrigel was diluted with serum-free medium pre-cooled at 4 °C to a final concentration of 1 mg/ml. Incubated on ice, 100 μl of diluted Matrigel was added vertically to the bottom center of the upper chamber of the chambers and incubated at 37 °C for 4 h to dry to a gelatinous consistency; 100 μl of digested serum-free cell suspension at a density of 1 × 10^5^ ml of each group was aspirated and added to the small chambers, and 800 μl of serum containing 10% serum was added to the bottom of the chambers. The lower chamber of the chamber was filled with 800 μl of medium containing 10% serum, and the incubation was continued for 48 h; the chambers were taken out, the upper chamber was wiped out, and the chambers were fixed with methanol for 20 min, stained with crystal violet for 20–30 min, and then photographed and counted under a microscope after the excess dye was washed off with PBS.

### Tunnel detection of apoptosis

The digested cells of HCT116 and HT29 were adjusted to 5 × 10^4^/ml and inoculated in 24-well plates. After 24 h, the drug-containing medium containing the corresponding final concentration of single and combination drugs was replaced according to the grouping, and the incubation was continued for 48 h. Incubation with 0.3% Triton XMel 100 PBS at room temperature for 5 min followed by washing once with PBS and fixing with 4% paraformaldehyde for 30 min. For TdT enzyme: fluorescent labeling solution = 1:9, samples were washed twice in PBS, TUNEL detection solution was added, samples were incubated for 60 min away from light, and samples were flushed with PBS three times. The expression of the positive signals of cellular regulation and death was observed under the fluorescence microscope.

### Cell cycle analysis

The digested cells of HCT116 and HT29 were adjusted to 5 × 10^4^/ml and inoculated into 6-well plates, respectively. After 24 h, the medium containing the corresponding final concentrations of single and combined drugs was changed according to the groups, and the incubation was continued for 48 h. Pre-cooled 70% ethanol was used to fix the cells overnight at −20 °C. A 1000 × *g* centrifuge with a 5 min centrifugation process was followed by a wash of the fine cell precipitates in pre-cooled PBS after the supernatant was discarded. The staining buffer (C solution) of 0.5 ml was mixed with 10 μl of PI (A solution) and 10 μl RNase A (B solution). Each sample was stained with PI staining solution prepared by 0.5 ml for 30 min, and flow cytometry was used to count the distribution of cell cycle phases.

### Measurement of intracellular reactive oxygen species levels

DNA damage was measured using the ROS assay kit (Beyotime, China, S0033S). The digested cells of HCT116 and HT29 were adjusted to 5 × 10^4^/ml and inoculated into 6-well plates. After 24 h, the drug-containing medium containing the corresponding final concentration of single and combined drugs was replaced by the group, and the incubation was continued for 48 h. Before loading the probes, the cells were diluted with H2DCFDA in serum-free culture medium according to the standard 1:1000 so that the final concentration was 10 μM. Prior to probe loading, H2DCFDA was diluted 1:1000 with serum-free culture medium to a final concentration of 10 μM. 0.5 ml of the diluted DCFH-DA working solution was added to each well of the cells, and the cells were incubated for 30 min at 37 °C in a cell culture incubator protected from light; the cells were washed 1–2 times with serum-free culture medium to remove the DCFH-DA that had not entered the cells completely; the DCFH-DA working solution was diluted with PBS. Hoechst33342 (100×) was added to the 1× working solution, the cells were covered and incubated at 37 °C for 10 min; the dye-containing culture medium was aspirated, and the cells were washed with PBS three times to observe the fluorescence under a fluorescence microscope.

### Measurement of oxidative DNA damage

DNA damage was determined by using a DNA damage detection kit (Beyotime, China, C2037S). The digested cells of HCT116 and HT29 were adjusted to 5 × 10^4^/ml and inoculated into 24-well plates, respectively. After 24 h, the drug-containing medium containing the corresponding final concentration of single and compound drugs was replaced by groups, and the incubation was continued for 48 h; the culture medium was aspirated, and the culture solution was washed once with PBS. Add fixation solution and fix for 10 min; remove the fixation solution and wash with washing solution three times for 3 min each time. Aspirate the residual liquid during each washing and keep the sample surface wet at the same time; add immunostaining sealing solution, sealing at room temperature for 10 min; aspirate the immunostaining sealing solution, add γ-H2AX murine monoclonal antibody, and incubate at 4 °C overnight; aspirate γ-H2AX murine monoclonal antibody, and wash with washing solution for three times; add anti-mouse 488, and incubate for 1 h at room temperature, and wash with washing solution for two times; add cytosolic staining solution (DAPI), room temperature staining for 5 min, aspirate the nucleus staining solution, wash with washing solution for three times, wash with culture solution for three times can be observed under the fluorescence microscope to observe the fluorescence.

### RT-qPCR

The digested cells of HCT116 and HT29 were adjusted to 5 × 10^4^/ml, inoculated in 6-well plates, respectively. Twenty four hours later, the drug-containing medium containing the corresponding final concentration of single and compound drugs was replaced according to groups. The concentration and purity of RNA were measured by a microspectrophotometer 48 h after incubation with Trizol (Allotype Gold, China; H10318). A reverse transcription kit (Beyotime, China; D7168M) was used to convert RNA samples into cDNA (Eppendorf, Germany; 5333 53658), followed by real-time quantitative polymerase chain reactions (AQ131-01) utilizing specific primers (Table 2), utilizing GAPDH as an internal control. The PCR reaction procedure is as follows: 95 °C pre-denaturation 3 min; 95 °C denaturation 30 s, 55 °C annealing 20 s, 72 °C extension 20 s, a total of 40 cycles (ABI, USA; 7500). The steps of the dissolution curve are as follows: 95 °C 15 s, 60 °C 15 s, 20 min heating, 95 °C 15 s.

**Table 2:** Primer sequence.

Primer	Sequence
GAPDH	F: 5′-GACAGTCAGCCGCATCTTCT-3′
	R: 5′- GCGCCCAATACGACCAAATC-3′
IL10	F: 5′-CATCAGGGGCTTGCTCTTGC -3′
	R: 5′-ACAGCTAGAAAGCGTGGTCA-3′
IL6	F: 5′-GTCCAGTTGCCTTCTCCCTG-3′
	R: 5′-TCTTCTCCTGGGGGTACTGG-3′
TNFα	F: 5′-CTCTCTGCCATCAAGAGCCC-3′
	R: 5′-CAGACTCGGCAAAGTCGAGA-3′
IL1β	F: 5′-TTTGAGTCTGCCCAGTTCCC-3′
	R: 5′-CTGACTGTCCTGGCTGATGG-3′
CXCL8	F: 5′-GAAGTTTTTGAAGAGGGCTGAGA-3′
	R: 5′-GGCACAGTGGAACAAGGACT-3′

### Western blotting

The digested cells of HCT116 and HT29 were adjusted to 5 × 10^4^ /ml and inoculated in 6-well plates, respectively. The drug-containing medium containing the corresponding final concentration of single and combination drugs was replaced by grouping after 24 h. Forty Eight hours of incubation were followed. Following 48 h of incubation, RIPA rapid lysate was used to extract total cellular proteins, and BCA protein analysis was performed to determine protein concentrations. SDS-PAGE separation gel was used to separate the proteins in the cells and transfer them to PVDF membranes (Millipore, USA; IPVH00010). In order to seal the PVDF membrane, 1 h of 5% skimmed milk was applied, then three times of TBST was applied, and incubated with P53(1:1000; Affinity; AF0879), P21(1:1000; Affinity; AF6290), PTGS2 (1:1000; Affinity; AF7003), IκBα (1:1000; Affinity; AF5002), AKT1/2/3 (1:1000; Affinity; AF6261), p-AKT1/2/3 (1:1000; Affinity; AF0016), NFκB1 (1:1000; Affinity; BF0466), p-NFκB1 (1:1000; Affinity; AF3219) were incubated. Protetintech’s GAPDH (1:2000) is used as a loading control. A secondary antibody conjugated to HPR (Solebo, China; SE134) was incubated with the immunoblots for 1 h at 37 °C. Beyotime chemiluminescent substrates (P0018S) were used for staining the blots and Tanon 5200 was used for image capture. GAPDH (Solebo, China; K106389) was used as a standard for determining the gray density of each protein band. Triplicates were performed on each assay.

### Statistical analysis

SPSS 21.0 software (IBM APSS Software) was used for statistical analysis. Results were reported as mean + standard deviation (SD), multiple group comparisons were analyzed using one-way ANOVA, and two group comparisons were analyzed using *t*-tests. *P* < 0.05 indicates that there is statistical significance between the two groups. The images were processed using GraphPad Prism 6.0 software (GraphPad Software).

## Supplementary information

Supplementary Material

## Data Availability

All data generated or analyzed during this study are included in this published article.
