# Supplementary material for: Oleanolic acid combined with aspirin plays antitumor roles in colorectal cancer via the Akt/NFκB/IκBα/COX2 pathway
Source: Cell Death Discov. 2024 Dec 18;10:504. doi: 10.1038/s41420-024-02223-9 (PMC11655652; doi:10.1038/s41420-024-02223-9)
Supplement: Supplementary file 1 — Supplementary Material [file 41420_2024_2223_MOESM1_ESM.pdf]

Supplementary material S1

Report of Human Cell Line Authentication

Delivery Date:2022-09-20

Analysis Date:2022-09-22

Ⅰ. Sample

Sample Name:‘XBRY0322-0436’, labeled as ‘CH1101’, was received on 2022-09-20.

Ⅱ.Method and Procedure

Sample DNA was extracted by Microread Genomic DNA Kit.

PCR was amplified with STR Multi-amplification Kit(Microreader^{TM}21 ID System).

PCR products were assayed with ABI 3730xl DNA Analyzer(Applied Biosystems®).

Data were analyzed using GeneMapperID-X software and then compared with the ATCC and DSMZ databases for reference matching.

Ⅲ. Results

The results of the negative and positive control match expectations.

The STR profiles of the test sample are clear and fine, which were shown in the attached table and figure.

Description of Multiple Alleles[1]:

Number of Multiple Alleles	Locus of Multiple Alleles
2	D16S539,vWA

Results of database compariso[n2] (the highest percent match to the query):

	ATCC	DSMZ	Test Cell							
Cell Name	HCT 116-Luc2 Colon Carcinoma Human	HCT 116	CH1101							
Cell No.	CCL-247-LUC2	HPACC	XBRY0322-0436							
Amelogenin	X	X,Y	X	X						
D5S818	10,11	10,11	10	11						
D13S317	10,12	10,12	10	12						
D7S820	11,12	11,12	11	12						
D16S539	11,13	11,13	11	13	14					
vWA	17,22	17,22	17	18	22	23				
TH01	8,9	8,9	8	9						
TPOX	8	8,8	8	8						
CSF1PO	7,10	7,10	7	10						
Matching[3]	100%	0.82								
Interpretation[4]	Identical	——								

Remarks:

1. More than or equal to three peaks at more than three loci (multiple alleles loci)suggests there may be cross-contamination from homologous species. Less than 3 multiple alleles loci might result from trisomy or mutation.

2. The test results was comparedagainst STR DNA profiles recorded in ATCC and DSMZ(DSMZ database includes data sets of 2455 cell lines from ATCC, DSMZ, JCRB and RIKEN).

3. The matchingvalue of ATCC database is (the number of shared alleles between query sample and database profile) / （total number of alleles in database profile） x 100%. The matchingvalue of DSMZ database is EV = (the number of generated peaks of test cell x2) / total number of peaks of (test cell + matched cell).

4. The interpretation of ATCC is based on “ANSI/ATCC ASN-0002-2011”, giving “Identical” to matches =100%, “related” to matches that≥ 80%, “require further investigation” to matches that from 79% to 56%, and “unrelated” to those matches less than 56%.As comparing with DSMZ, giving “Identical” to only when EV=1.

IV. Conclusions

CH1101:

① No cross-contamination of other human cell line is found.

② The submitted profile has a100% match for the following ATCC human cell line(s) in ATCC STR database (8 core loci plus Amelogenin): HCT 116-Luc2 Colon Carcinoma Human.

③ Between the submitted profile and HCT 116 ,the STR matching rate（EV value） is 0.82 in DSMZ STR database.

Table: STR profiles ofCH1101cell line

Cell lineCH1101（Fig.XBRY0322-0436）								
Marker	Allele 1	Allele 2	Allele 3	Allele 4	Allele 5	Allele 6	Allele 7	Allele 8
D19S433	12	13						
D5S818	10	11						
D21S11	29	30						
D18S51	16	17						
D6S1043	13	13						
AMEL	X	X						
D3S1358	12	17	19					
D13S317	10	12						
D7S820	11	12						
D16S539	11	13	14					
CSF1PO	7	10						
PentaD	9	13						
D2S441	11	12						
vWA	17	18	22	23				
D8S1179	12	14						
TPOX	8	8						
PentaE	13	14						
TH01	8	9						
D12S391	17	21	22					
D2S1338	16	16						
FGA	18	23						

Figure1: STR profiles ofCH1101cell line

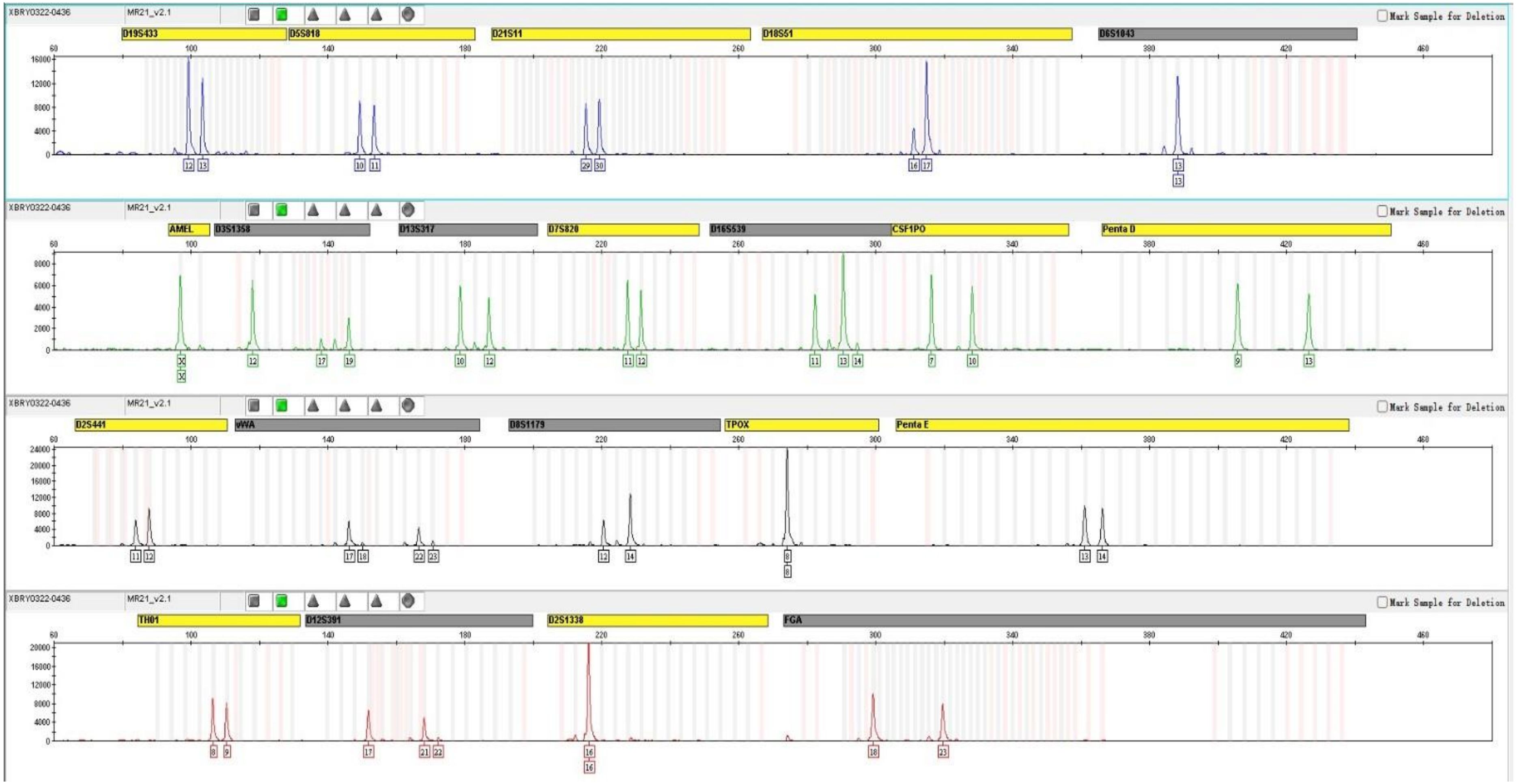

Figure2: Result of STR matching analysis in ATCC STR database

Show per page

Showing **1-4** of **4**

24

Add to Cart	%Match	ATCC® Number	Designation	D5S818	D13S317	D7S820	D16S539	vWA	TH01	AMEL	TPOX	CSF1PO
	100.0	CCL-247-LUC2	HCT 116-Luc2 Colon Carcinoma Human	10,11	10,12	11,12	11,13	17,22	8,9	X	8	7,10
	89.0	CCL-247	HCT 116Colon CarcinomaHuman	10,11	10,12	11,12	11,13	17,22	8,9	X,Y	8,9	7,10
	88.0	CCL-247EMT-MCB	HCT-116 Vimentin RFP Reporter Lung Carcinoma Human	10,11	10,13	11,12	11,13,14	20,22	8,9	X	8	7,10
	88.0	CRL-2780	ATRFLOXColon CarcinomaHuman	10,12	10,12	11,12	11,13	17,22	8,9	X	8	7,9

ADD TO CART EXPORT TO EXCEL

Show per page

24

Figure3: Result of STR matching analysis in DSMZ STR database

STR Profile Search

Help

The human STR profile database includes data sets of 2455 cell lines from ATCC, DSMZ, JCRB and RIKEN.

Refine search | Start new search

Similarity	Cell line	Source	D5S818			D13S317			D7S820		D16S539				vWA				TH01		TPOX		CSF1PO		Amelogenin		D3S135	
?	Your query		10	11		10	12		11	12	11	13	14		17	18	22	23	8	9	8	8	7	10	X	X		
82.1 %	HCT 116	HPACC	10	11		10	12		11	12	11	13			17	**22**			8	9	8	8	7	10	X	**Y**	**12**	[illegible]
82.1 %	HCT 116	KCLB Korea 10247	10	11		10	12		11	12	11	13			17	**22**			8	9	8	8	7	10	X	**Y**	**12**	[illegible]
76.9 %	HCT 116	CCL-247	10	11		10	12		11	12	11	13			17	**22**			8	9	8	**9**	7	10	X	**Y**	**12**	[illegible]
76.9 %	ATRFLOX [Mutat...	CRL-2780	10	**12**		10	12		11	12	11	13			17	**22**			8	9	8	8	7	**9**	X	X		
76.9 %	ACJ Cells No. 41	STRJ0016	10	11		10	**11**		11	12	11	13			17	**17**			8	9	8	8	7	**9**	X	X		
74.4 %	HCT-116	DSMZ: ACC-581	10	11		10	12		11	12	11	13	**12**	**14**	17	**22**	**21**	23	8	9	8	**9**	7	10	X	**Y**	**12**	[illegible]
61.9 %	ACJ Cells No. 9	STRJ0006	**9**	**10**	**11**	10	**11**	**12**	11	12	11	**12**	**13**		17	**21**			8	9	8	8	7	10	X	X		
61.5 %	A3/KAW	JCRB0101	10	11		10	12		11	12	**10**	**10**			17	**17**			**7**	9	8	8	**11**	**12**	X	X		
56.4 %	CL-14	DSMZ: ACC-504	**11**	11		**8**	12		11	12	11	13			**16**	18			**6**	**8**	8	8	**12**	**12**	X	X	**17**	[illegible]
56.4 %	CCD-1001Sk	CRL-1987	10	11		**11**	12		**9**	**11**	**10**	13			17	18			8	9	8	8	**11**	**15**	X	**Y**		

Supplementary material S2

Report of Human Cell Line Authentication

Delivery Date:2022-09-20

Analysis Date:2022-09-22

Ⅰ. Sample

Sample Name:'XBRY0322-0433', labeled as 'CH1235', was received on 2022-09-20.

Ⅱ.Method and Procedure

Sample DNA was extracted by Microread Genomic DNA Kit.

PCR was amplified with STR Multi-amplification Kit(Microreader™21 ID System).

PCR products were assayed with ABI 3730xl DNA Analyzer(Applied Biosystems®).

Data were analyzed using GeneMapperID-X software and then compared with the ATCC and DSMZ databases for reference matching.

Ⅲ. Results

The results of the negative and positive control match expectations.

The STR profiles of the test sample are clear and fine, which were shown in the attached table and figure.

Description of Multiple Alleles[1]:

Number of Multiple Alleles	Locus of Multiple Alleles
0	——

Results of database compariso[n2] (the highest percent match to the query):

	ATCC	DSMZ	Test Cell							
Cell Name	HT-29 Colon Adenocarcinoma Human	HT-29	CH1235							
Cell No.	HTB-38	HTB-38	XBRY0322-0433							
Amelogenin	X	X,X	X	X						
D5S818	11,12	11,12	11	12						
D13S317	11,12	11,12	11	12						
D7S820	10	10,10	10	10						
D16S539	11,12	11,12	11	12						
vWA	17,19	17,19	17	19						
TH01	6,9	6,9	6	9						
TPOX	8,9	8,9	8	9						
CSF1PO	11,12	11,12	11	12						
Matching[3]	100%	1								
Interpretation[4]	Identical	Identical								

Remarks:

1. More than or equal to three peaks at more than three loci (multiple alleles loci)suggests there may be cross-contamination from homologous species. Less than 3 multiple alleles loci might result from trisomy or mutation.

2. The test results was comparedagainst STR DNA profiles recorded in ATCC and DSMZ(DSMZ database includes data sets of 2455 cell lines from ATCC, DSMZ, JCRB and RIKEN).

3. The matchingvalue of ATCC database is (the number of shared alleles between query sample and database profile) / （total number of alleles in database profile） x 100%. The matchingvalue of DSMZ database is EV = (the number of generated peaks of test cell x2) / total number of peaks of (test cell + matched cell).

4. The interpretation of ATCC is based on “ANSI/ATCC ASN-0002-2011”, giving “Identical” to matches =100%, “related” to matches that≥ 80%, “require further investigation” to matches that from 79% to 56%, and “unrelated” to those matches less than 56%.As comparing with DSMZ, giving “Identical” to only when EV=1.

IV. Conclusions

CH1235:

①No cross-contamination of other human cell line is found.

②The submitted profile has a100% match for the following ATCC human cell line(s) in ATCC STR database (8 core loci plus Amelogenin): HT-29 Colon Adenocarcinoma Human.

③ Between the submitted profile and HT-29 ,the STR matching rate（EV value）is 1 in DSMZ STR database.

Table: STR profiles ofCH1235cell line

Cell lineCH1235（Fig.XBRY0322-0433）								
Marker	Allele 1	Allele 2	Allele 3	Allele 4	Allele 5	Allele 6	Allele 7	Allele 8
D19S433	14	14						
D5S818	11	12						
D21S11	29	30						
D18S51	13	13						
D6S1043	12	14						
AMEL	X	X						
D3S1358	15	17						
D13S317	11	12						
D7S820	10	10						
D16S539	11	12						
CSF1PO	11	12						
PentaD	11	13						
D2S441	11	11						
vWA	17	19						
D8S1179	10	16						
TPOX	8	9						
PentaE	14	16						
TH01	6	9						
D12S391	18.3	21						
D2S1338	19	23						
FGA	20	22						

Figure1: STR profiles ofCH1235cell line

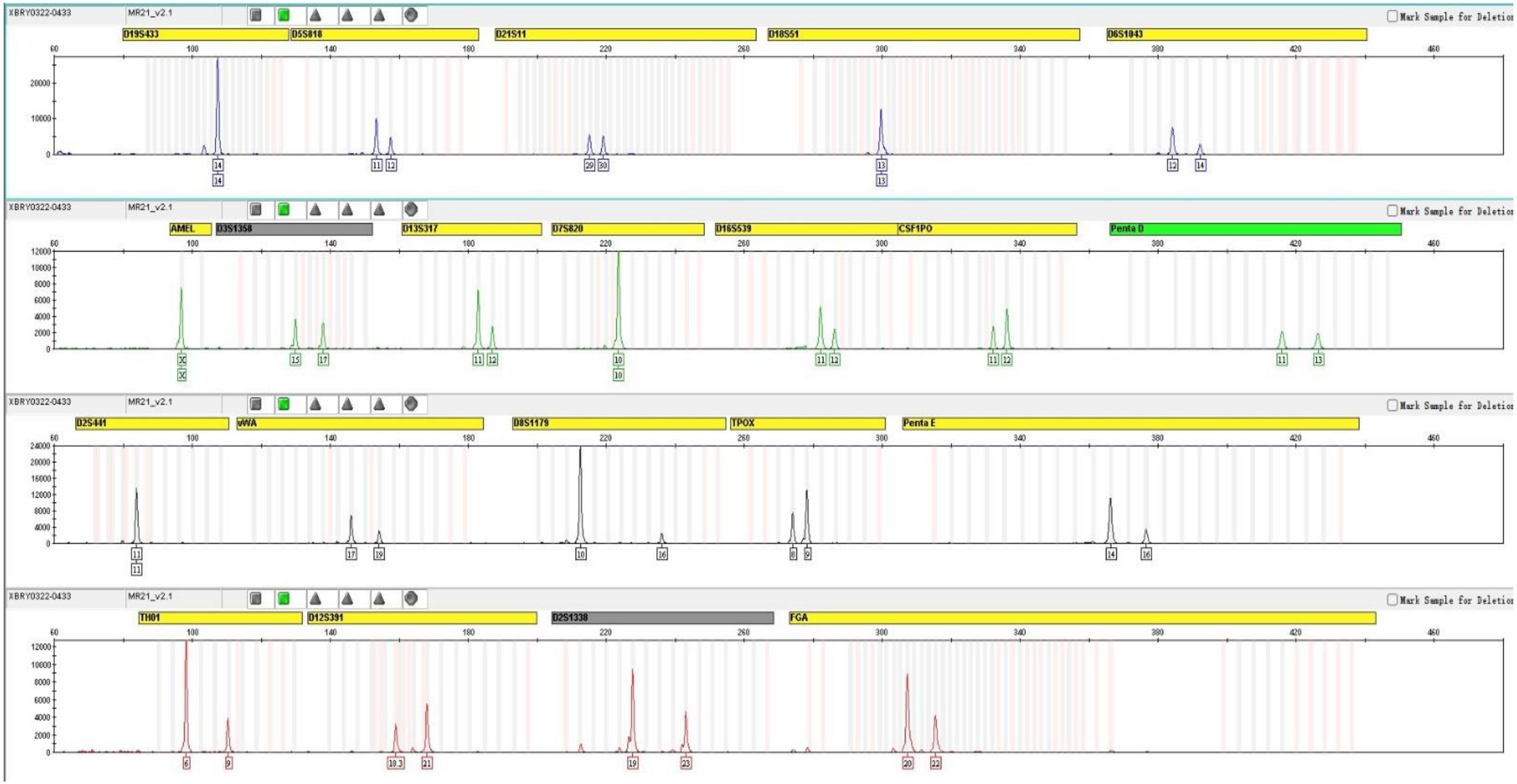

Figure2: Result of STR matching analysis in ATCC STR database

Showing **1-8** of **8**

Show per page

24

Add to Cart	%Match	ATCC® Number	Designation	D5S818	D13S317	D7S820	D16S539	vWA	TH01	AMEL	TPOX	CSF1PO
	100.0	CCL-218	WiDrColon AdenocarcinomaHuman	11,12	11,12	10	11,12	17,19	6,9	X	8,9	11,12
	100.0	HTB-38	HT-29Colon AdenocarcinomaHuman	11,12	11,12	10	11,12	17,19	6,9	X	8,9	11,12
	92.0	HTB-161	NIH:OVCAR-3Ovarian AdenocarcinomaHuman	11,12	12	10	12	17	9,9.3	X	8	11,12
	89.0	HTB-56	Calu-6Anaplastic CarcinomaHuman	11	11	10	13	17	9	X	8	12
	86.0	PDM-116	HCM-BROD-0045-C16 Cancer Model Metastatic Gastric adenocarci	11	11,12	9,10	11,12	16,17,19	6	X	8	11
	83.0	CRL-5902	NCI-H1876Lung CarcinomaHuman	11	12	10,12	12	17	6	X,Y	8,9	11
	83.0	CRL-5903	NCI-H1882Lung CarcinomaHuman	11	12	10,12	12	17	6	X,Y	8,9	11
	82.0	HTB-35	SiHaCervical CarcinomaHuman	9	11	10	12	14,17	6,9	X	8	12

ADD TO CART EXPORT TO EXCEL

Show per page

24

Figure3: Result of STR matching analysis in DSMZ STR database

STR Profile Search

Help

The human STR profile database includes data sets of 2455 cell lines from ATCC, DSMZ, JCRB and RIKEN.

Refine search | Start new search

Similarity	Cell line	Source	D5S818		D13S317		D7S820		D16S539		vWA		TH01		TPOX		CSF1PO		Amelogenin	
?	Your query		11	12	11	12	10	10	11	12	17	19	6	9	8	9	11	12	X	X
100 %	HT-29	DSMZ: ACC-299	11	12	11	12	10	10	11	12	17	19	6	9	8	9	11	12	X	X
100 %	WiDr	CCL-218	11	12	11	12	10	10	11	12	17	19	6	9	8	9	11	12	X	X
100 %	MV522	CRL-2519	11	12	11	12	10	10	11	12	17	19	6	9	8	9	11	12	X	X
100 %	MV522/MDR1	CRL-2520	11	12	11	12	10	10	11	12	17	19	6	9	8	9	11	12	X	X
100 %	USC-HNI	CRL-3026	11	12	11	12	10	10	11	12	17	19	6	9	8	9	11	12	X	X
100 %	HT29	HPACC	11	12	11	12	10	10	11	12	17	19	6	9	8	9	11	12	X	X
100 %	WiDr	HPACC	11	12	11	12	10	10	11	12	17	19	6	9	8	9	11	12	X	X
100 %	HT-29	HTB-38	11	12	11	12	10	10	11	12	17	19	6	9	8	9	11	12	X	X
100 %	WiDr	IFO50043	11	12	11	12	10	10	11	12	17	19	6	9	8	9	11	12	X	X
100 %	WiDr	JCRB0224	11	12	11	12	10	10	11	12	17	19	6	9	8	9	11	12	X	X

HCT116

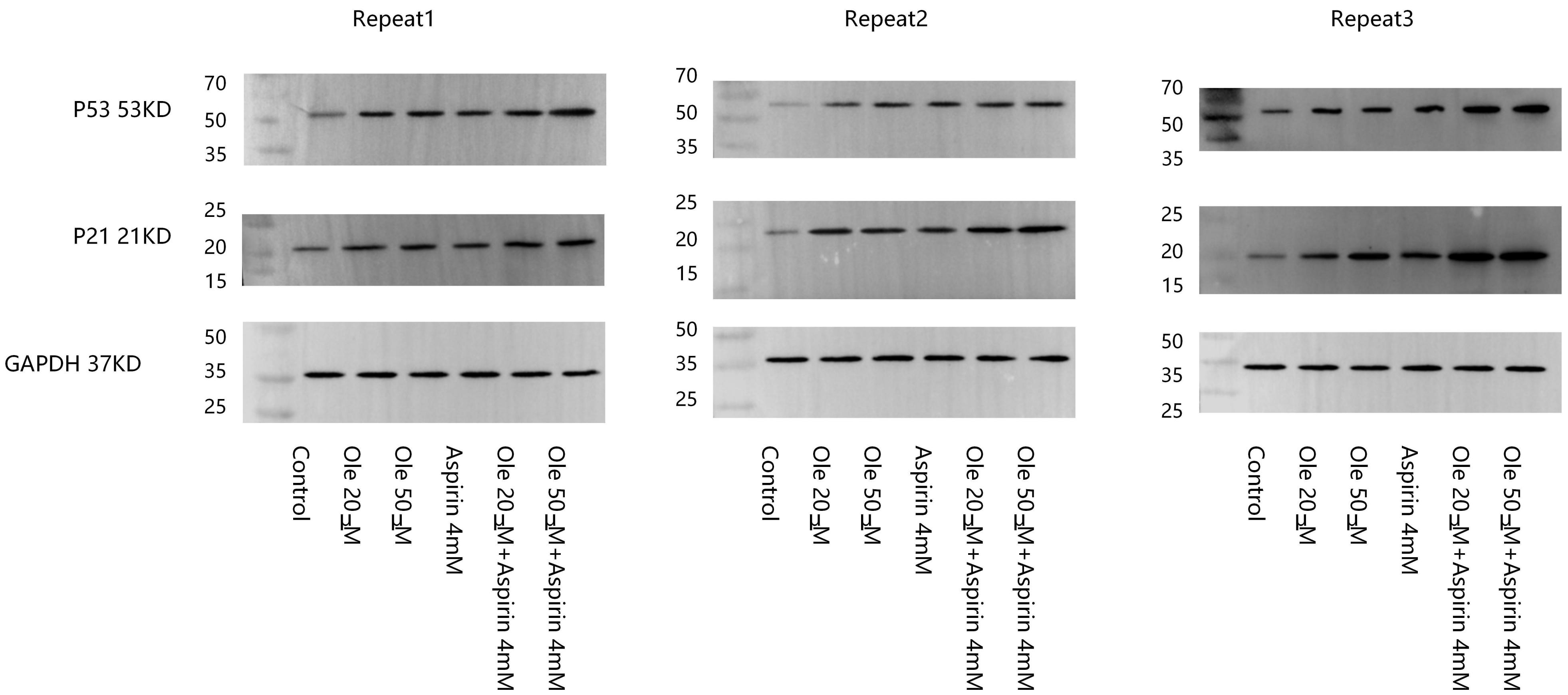

Figure S3. Three repetitions of the original plot of the HCT116 cell western blot assay in Figure 6C.

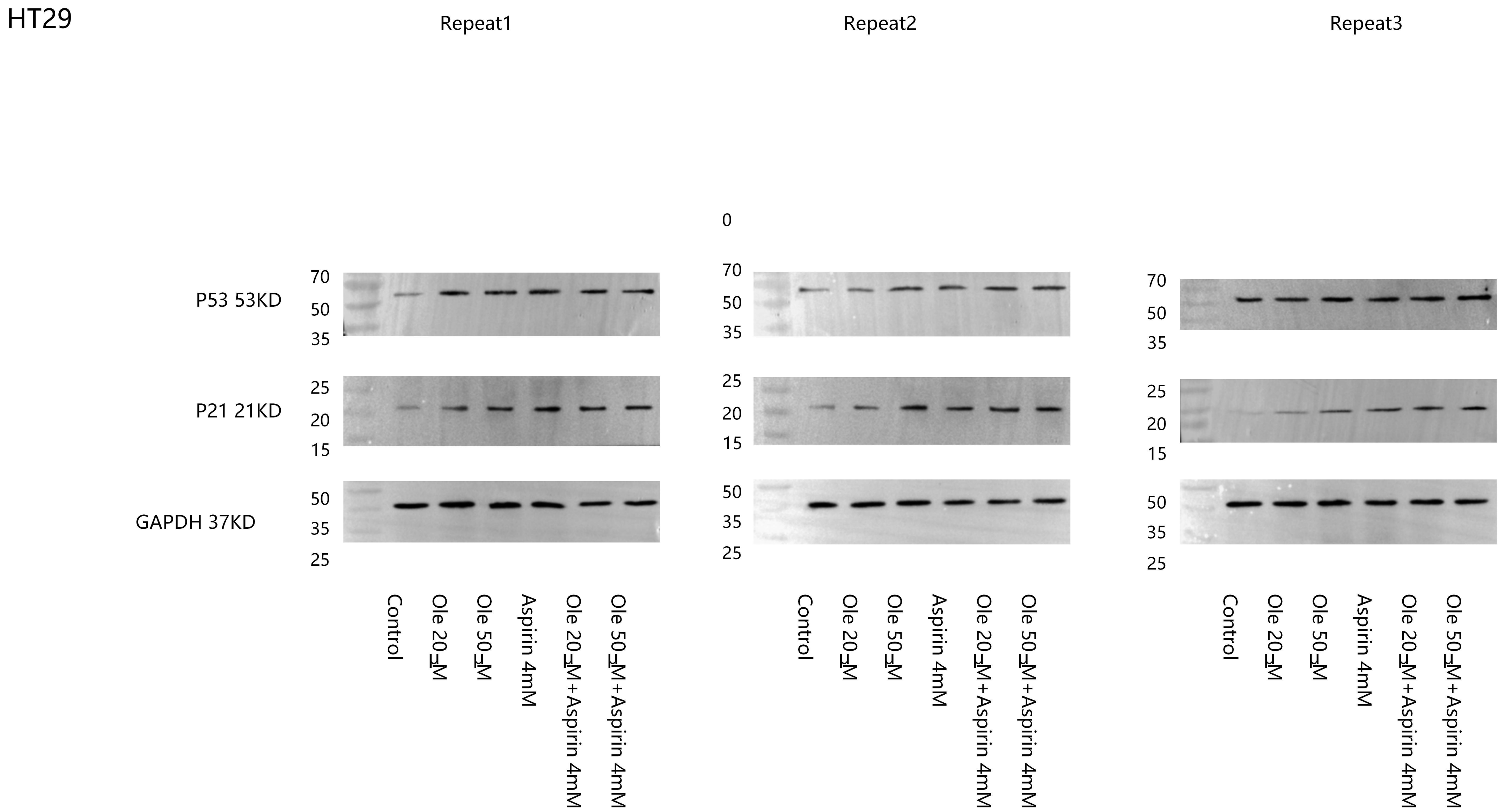

Figure S4. Three repetitions of the original plot of the HT29 cell western blot assay in Figure 6C.

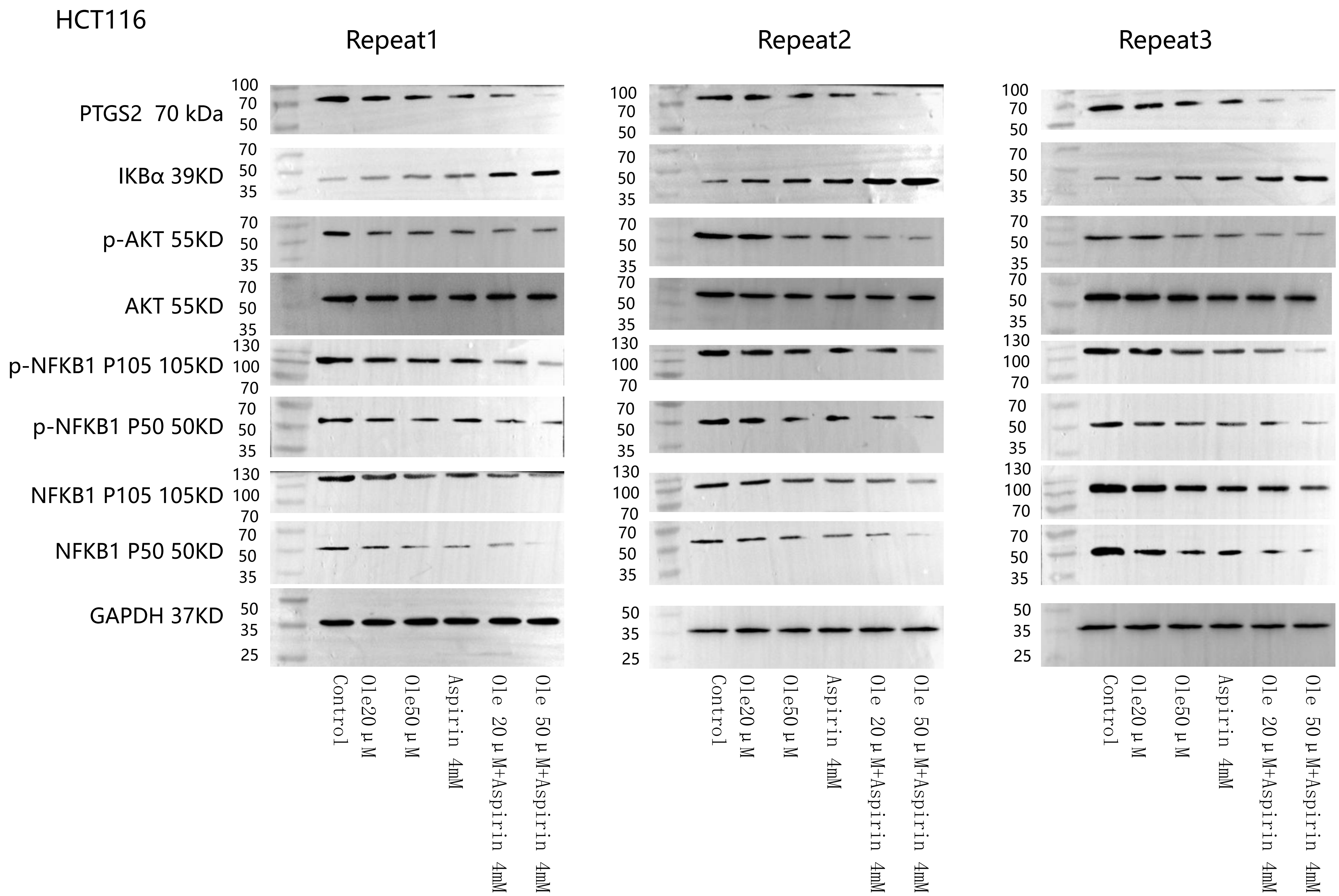

Figure S5. Three repetitions of the original plot of the HCT116 cell western blot assay in Figure 7.
